# Innate immunity in peripheral tissues is differentially impaired under normal and endotoxic conditions in aging

**DOI:** 10.3389/fimmu.2024.1357444

**Published:** 2024-08-16

**Authors:** Ji Yeon Noh, Hye Won Han, Da Mi Kim, Erin D. Giles, Yuhua Z. Farnell, Gus A. Wright, Yuxiang Sun

**Affiliations:** ^1^ Department of Nutrition, Texas A&M University, College Station, TX, United States; ^2^ School of Kinesiology, University of Michigan, Ann Arbor, MI, United States; ^3^ Department of Poultry Science, Texas A&M University, College Station, TX, United States; ^4^ Department of Veterinary Pathobiology, Texas A&M University, College Station, TX, United States; ^5^ Department of Biochemistry & Biophysics, Texas A&M University, College Station, TX, United States

**Keywords:** aging, inflammaging, immunometabolism, innate immunity, macrophages, monocytes, neutrophils

## Abstract

Chronic low-grade inflammation is a hallmark of aging, aka “inflammaging”, which is linked to a wide range of age-associated diseases. Immune dysfunction increases disease susceptibility, and increases morbidity and mortality of aging. Innate immune cells, including monocytes, macrophages and neutrophils, are the first responders of host defense and the key mediators of various metabolic and inflammatory insults. Currently, the understanding of innate immune programming in aging is largely fragmented. Here we investigated the phenotypic and functional properties of innate immune cells in various peripheral tissues of young and aged mice under normal and endotoxic conditions. Under the steady state, aged mice showed elevated pro-inflammatory monocytes/macrophages in peripheral blood, adipose tissue, liver, and colon. Under lipopolysaccharide (LPS)-induced inflammatory state, the innate immune cells of aged mice showed a different response to LPS stimulus than that of young mice. LPS-induced immune responses displayed differential profiles in different tissues and cell types. In the peripheral blood, when responding to LPS, the aged mice showed higher neutrophils, but lower pro-inflammatory monocytes than that in young mice. In the peritoneal fluid, while young mice exhibited significantly elevated pro-inflammatory neutrophils and macrophages in response to LPS, aged mice exhibited decreased pro-inflammatory neutrophils and variable cytokine responses in macrophages. In the adipose tissue, LPS induced less infiltrated neutrophils but more infiltrated macrophages in old mice than young mice. In the liver, aged mice showed a more robust increase of pro-inflammatory macrophages compared to that in young mice under LPS stimulation. In colon, macrophages showed relatively mild response to LPS in both young and old mice. We have further tested bone-marrow derived macrophages (BMDM) from young and aged mice, we found that BMDM from aged mice have impaired polarization, displaying higher expression of pro-inflammatory markers than those from young mice. These data collectively suggest that innate immunity in peripheral tissues is impaired in aging, and the dysregulation of immunity is tissue- and cell-dependent. Our findings in the rodent model underscore the complexity of aging immunity. Further investigation is needed to determine whether the immune profile observed in aged mice is applicable in age-associated diseases in humans.

## 1 Introduction

The aging population is on the rise worldwide. Increasing health span is in the forefront of aging research and is a challenging task for the global health care system (1). Aging is a major risk factor for chronic illnesses (2). Inflammaging is the condition of age-associated chronic low-grade inflammation (3), which is linked to the development and progression of various age-related chronic diseases such as obesity, type 2 diabetes, cardiovascular disease, and inflammatory bowel disease (4). There is clear evidence that elevated inflammatory cytokines, such as TNFα, IL-β, and IL-6, are correlated with high mortality rates in old individuals (5–8).

The immune system is critical for survival, as it defends the host from various inflammatory stimuli such as pathogens and mutated endogenous substances. Immune dysfunction in aging increases susceptibility to disease; it diminishes vaccination responses and contributes to high morbidity and mortality in the elderly (9). To date, the T and B cells in aging have been well-documented (10, 11), whereas the innate immunity in aging remains understudied. Given that innate immunity is a first-line defense and plays a critical role in initiation and resolution of inflammation (12), it is of paramount importance to understand innate immunity in aging, which will have important implications in age-associated diseases and healthspan.

Innate immune cells are highly plastic, showing dynamic response to inflammatory stimuli in order to rapidly adapt to the microenvironment and perform specific functions; this is referred to polarization. The polarization of innate immune cells leads to different functional outcomes, which is traditionally described as a dichotomous concept; e.g., the M1-M2 paradigm of macrophage polarization (13–16). This concept is widely applied *in vitro*, but evidences now suggest that tissue macrophages are heterogeneous and present across a wide spectrum that is not restricted to the dichotomous M1-M2 characterization (17–19).

It has been shown that aging impacts the programming of innate immune cells and contributes to inflammaging and age-associated diseases (20–22). However, it is largely unknown if age-associated innate immune programming is tissue- and/or cell-specific. Here we uncovered the phenotypic and functional properties of innate immune cells, particularly neutrophils and macrophages, in a wide range of peripheral tissues in young and aged mice under both steady state and LPS-induced inflammatory state. We assessed properties of neutrophils and macrophages, as well as inflammatory cytokines produced by these immune cells, to assess functional outcomes using flow cytometry. The observed tissue-specific differential changes in these immune cells underscore the dysregulated immune responses in aging, thus increasing the susceptibility to the development of age-associated chronic diseases.

## 2 Materials and methods

### 2.1 Animal model

Animal experiments were conducted with C57BL/6J background adult male young (4-to-7-month-old) and aged (17-to-27-month-old) mice. All experimental animal protocols were approved by the Texas A&M University Institutional Animal Care and Use Committee (protocol code: IACUC 2022-0180, approval date: October 4, 2022). The mice were bred and housed under 12 h light-dark cycles, at 23 ± 1°C, with *ad libitum* access to water and semi-purified diet (Harlan-Teklad, Madison, WI, USA) which provided 58% kcal from carbohydrates, 24% kcal from protein, and 18% kcal from fat. To determine the inflammatory response, young (4-to-7-month-old) and aged (17-to-27-month-old) male mice received a single *i.p.* injection of lipopolysaccharide (LPS) 0.75 mg/kg and tissues were collected 6 hours after LPS exposure. At termination, the mice were euthanized using isoflurane anesthesia. The data from with and without LPS treatment groups were collected together within the same age group.

### 2.2 Tissue collection, digestion, and single cell preparation

#### 2.2.1 Peripheral blood

Mice were anesthetized using isoflurane and then whole blood was collected from retro-orbital into EDTA-coated MiniCollect tubes (Greiner, USA). For flow cytometry, 50 μL of whole blood was incubated with BD Fc block™ (CD16/32 clone, BD Bioscience, 553141) for 15 min on ice followed by surface marker staining with CD45-BV510 (Biolegend, 103138), CD11b-PE (Biolegend, 101208), Ly6C-eFluor450 (Thermofisher, 48-5932-82), Ly6G-BV785 (Biolegend, 127645), CD115-APC (Biolegend, 135509), CCR2-FITC (Biolegend, 150607) for 30 min. Red blood cells were then lysed with FACS Lysing Solution (BD Bioscience, 349202) in 1:20 ratio for 20 min at room temperature followed by 2% PFA for 30 min on ice. AccuCheck counting beads (Thermofisher, PCB100) were added to samples before data acquisition to enable the computation of absolute cell counts in PBMC, as per the manufacturer’s instructions. Fluorescence was detected using BD LSR Fortessa™ X-20 (BD Bioscience, San Jose, CA, USA) and/or Cytek® Aurora (Cytek®, Fremont, CA, USA). The gating strategy is noted in **Supplementary Figure 1A**. Briefly, neutrophils were gated as CD45^+^CD11b^+^Ly6G^+^ and monocytes were gated as CD45^+^CD11b^+^CD115^+^. Then, monocyte subpopulations were further distinguished by differential expression of Ly6C. We confirmed that the Ly6C^hi^ subset expressed high levels of CCR2, whereas Ly6C^inter^and Ly6C^low^ subsets had low expression of CCR2.

#### 2.2.2 Peritoneal fluid cells

Peritoneal fluid was collected as described previously (23). Briefly, 3 ml of ice-cold phosphate-buffered saline (PBS) containing 2% fetal bovine serum (FBS) was injected into the peritoneum. After gentle shaking for 1 min, peritoneal fluid was collected with an 18G syringe needle. The samples were then centrifuged at 500 g for 5 mins at 4°C. To lyse red blood cells, ACK lysing buffer (Fisher scientific, 50-101-9080) was added to the cell pellets and incubated for 5 min. Then, cells were collected by centrifugation at 500 g for 10 min at 4°C followed by washing twice with PBS. Golgi plug (BD Bioscience) was added to the cells for 4 hours followed by antibody staining as described in the Flow cytometry staining and cell analysis section below. The surface staining markers were, CD45-eFluor450 (eBioscience, 50-112-9409), Ly6G-BV785 (Biolegend, 127645), CD11b-APC-Cy7 (Biolegend, 101226), F4/80-PE-Cy7 (eBioscience, 25-4801-82), CD38-PE (BD Bioscience, 553764), and CD206-Alexa Fluor 488 (Biolegend, 141710). Additionally, to assess functional outcome more directly, intracellular antibodies were used to test cytokine expression in specific cells. TNFα-PE-Dazzle594 (Biolegend, 506345), IL-1β-PE (Thermofisher, 12-7114-80), iNOS-Alexa Fluor 488 (Thermofisher, 53-5920-80) and Arg1-Alexa Fluor700 (Thermofisher, 56-3697-82) were used to stain cells for 30 min on ice. Cells were then washed with the perm wash buffer. Data were acquired using a BD LSR Fortessa™ X-20 (BD Bioscience, San Jose, CA, USA) and/or Cytek® Aurora (Cytek®, Fremont, CA, USA). The flow cytometry gating strategy is detailed in **Supplementary Figure 2**. Briefly, peritoneal neutrophils were defined as CD45^+^CD11b^+^Ly6G^+^. The intracellular markers (TNFα, IL-1β, and iNOS) were evaluated from neutrophil population (CD45^+^CD11b^+^Ly6G^+^). Peritoneal macrophages were defined as CD45^+^CD11b^+^Ly6G^-^F4/80^+^. The surface markers (CD38 and CD206) and intracellular markers (TNFα, IL-1β, iNOS, and Arg1) were evaluated from macrophage population (CD45^+^CD11b^+^Ly6G^-^F4/80^+^).

#### 2.2.3 Stromal vascular fraction from epididymal adipose tissue

Stromal vascular fraction was collected from epididymal adipose tissue as described previously (24). In brief, approximately 1 g of epididymal fat was minced using scissors and incubated in digestion buffer (1 mg/ml collagenase type I in RPMI medium) for 30 min at 37°C in a shaking water bath. The samples were filtered with 70 μm cell strainers to remove undigested tissue. After centrifuging at 1,700 g for 3 min, the cell pellets were collected and incubated with 4 ml of ACK lysing buffer (Fisher scientific, 50-101-9080) on ice for 10 min. The samples were then centrifuged at 1,700 g for 3 mins and the pellet of stromal vascular fraction (SVF) was re-suspended in 1 ml of PBS with 2% FBS. The antibody staining was described in the Flow cytometry staining and cell analysis section below. Surface markers were stained using CD45-BV510 (Biolegend, 557235), Ly6G-AlexaFluor 700 (Biolegend, 127622), CD11b-APC-cy7 (Biolegend, 101226), F4/80-PE-cy7 (eBioscience, 25-4801-82), and CX3CR1-BV421 (Biolegend, 149023) for 30 min on ice. The cells were washed with FACS buffer (1xPBS and 2% FBS) and then stained with 7AAD (BD Bioscience) before data collection. The data was acquired using MoFlo Astrios EQ (Beckman Coulter Life Sciences, Indianapolis, IN, USA) and/or Cytek® Aurora (Cytek®, Fremont, CA, USA). The flow gating strategy and representative plots are shown in **Supplementary Figure 3**. Neutrophils were gated as CD45^+^CD11b^+^Ly6G^+^. Infiltrating macrophages were defined as CD45^+^CD11b^+^Ly6G^-^F4/80^hi^ and resident macrophages were defined as CD45^+^CD11b^+^Ly6G^-^F4/80^low^. The surface markers (CD38 and CD206) were evaluated from the macrophage population (CD45^+^CD11b^+^Ly6G^-^ F4/80^+^).

#### 2.2.4 Liver

The gallbladder was carefully removed first, and then the liver was dissected. A portion of liver tissue was digested into single cell suspension using the Liver Dissociation Kit for mouse (Miltenyi Biotec, Auburn, CA, USA, 130-105-807) following the manufacturer’s instructions. Briefly, liver was rinsed with DMEM then transferred into MACS C tubes containing a dissociation enzyme mix, which was pre-heated for 30 min at 37°C. The samples were then digested using GentleMACS™ Octo Dissociator with heaters (Miltenyi Biotec, Auburn, CA, USA). The cell suspensions were filtered with 100 μm strainers, then centrifuged at 300 g for 10 min. Resuspended cells were stained with antibodies as described in the Flow cytometry staining and cell analysis section below. The antibodies used for cell surface staining were CD45-PerCP (BD Bioscience, 557235), Ly6G-BV785 (Biolegend, 127645), F4/80-PE-Cy7 (eBioscience, 25-4801-82), CD11b-APC-Cy7(Biolegend, 101226), CD38-PE (BD Bioscience, 553764), and CD206-BV650 (Biolegend, 141723). The intracellular staining antibodies were TNFα-Alexa Fluor 700 (BD Bioscience, 558000), IL-1β-PE (Thermofisher, 12-7114-80), and iNOS-Alexa Fluor 488 (Thermofisher, 53-5920-80). The data was acquired using Cytek® Aurora (Cytek®, Fremont, CA, USA). The flow gating strategy and representative plots are shown in **Supplementary Figure 4**. Neutrophils were selected as CD45^+^CD11b^+^Ly6G^+^. The intracellular markers (TNFα, IL-1β, and iNOS) were evaluated from neutrophil population (CD45^+^CD11b^+^Ly6G^+^). Infiltrating macrophages were defined as CD45^+^CD11b^hi^Ly6G^-^F4/80^low^ and resident macrophages were defined as CD45^+^CD11b^hi^Ly6G^-^ F4/80^hi^. The surface markers (CD38 and CD206) and intracellular markers (TNFα, IL-1β, and iNOS) were evaluated from macrophage population (CD45^+^CD11b^+^Ly6G^-^F4/80^+^).

#### 2.2.5 Colon

The colon was first washed with ice-cold phosphate-buffered saline, then cut longitudinally in the middle. Half colon was digested to obtain a single-cell suspension using the Lamina Propria Dissociation Kit for mouse (Miltenyi Biotec, Auburn, CA, USA, 130-097-410) according to the manufacturer’s instructions. In brief, the colon samples were washed with 1x HBSS without Ca^2+^ and Mg^2+^ containing 10 mM HEPES, 5mM EDTA and 5% FBS, and then the samples were digested by the enzyme mix from the Lamina Propria Dissociation kit (Miltenyi Biotec, Auburn, CA, USA) using GentleMACS™ Octo Dissociator with heaters (Miltenyi Biotec, Auburn, CA, USA). The cell suspensions were filtered with 100 μm strainers. The single-cell suspensions were then incubated with Golgi plug (BD Bioscience) for 4 hours then stained with fluorescence-conjugated antibodies of targeted markers for flow cytometry as described in the Flow cytometry staining and cell analysis section below. Surface marker antibodies used were CD45-FITC (eBioscience, 11-0451-82), CD11b-APC-cy7 (Biolegend, 101226), Ly6G-BV785 (Biolegend, 127645), Ly6C-eFluor450 (eBioscience,48-5932-82), MHCII-Alexa Fluor700 (Biolegend, 107622), CD64-Alexa Fluor647 (Biolegend, 139322), and CD206-PerCP-cy5.5 (Biolegend, 141716). Intracellular markers used were TNFα-PE-Dazzle594 (Biolegend, 506345) and IL-1β-PE (Thermofisher, 12-7114-80). The data were acquired using MoFlo Astrios EQ (Beckman Coulter Life Sciences, Indianapolis, IN, USA) and/or Cytek® Aurora (Cytek®, Fremont, CA, USA). The flow gating strategy and representative plots are shown in **Supplementary Figure 5**. Briefly, neutrophils were identified as CD45^+^CD11b^+^Ly6G^+^. The intracellular markers (TNFα and IL-1β) were evaluated from neutrophil population (CD45^+^CD11b^+^Ly6G^+^). The monocyte/macrophage population was selected as CD45^+^CD11b^+^Ly6G^-^CD64^+^. The surface markers (Ly6C and CD206) and intracellular markers (TNFα and IL-1β) were evaluated from monocytes/macrophage population (CD45^+^CD11b^+^Ly6G^-^CD64^+^).

#### 2.2.6 Bone marrow-derived macrophage differentiation and polarization

Bone marrow cells were collected from mouse femurs and tibias as previously described (25). 4×10^6^ cells were seeded in a 10 cm plate with differentiation medium (RPMI 1640 medium supplemented with 10 ng/mL macrophage colony-stimulating factor (M-CSF) (Peprotech, 315-02-250UG), 10% fetal bovine serum, 100U/ml penicillin, and 100 µg/ml streptomycin). The cells were incubated in a humidified incubator at 37°C with 5% CO_2_ for 7 days. On day 7, 2×10^5^ cells/well were seeded to 96 well plates and treated with LPS 100 ng/mL for either 4 or 24 hours, and then incubated with Golgi Plug™ (BD Bioscience) for 4 hours to enhance the detectability of cytokine-producing cells in subsequent immunofluorescent staining. The cells were subsequently stained as described in the Flow cytometry staining and cell analysis section below. The surface staining antibodies were CD11b-eFluor450 (eBioscience, 48-0112-82), F4/80-PE-Cy7 (eBioscience, 25-4801-82), and CD38-PE (BD Bioscience, 553764) for 30 min on ice. The intracellular staining antibodies used were TNFα-Alexa Fluor700 (BD Bioscience, 558000), IL-1β-PE (Thermofisher, 12-7114-80), and iNOS-Alexa Fluor 488 (Thermofisher, 53-5920-80). The data were acquired by BD LSR Fortessa™ X-20 (BD Bioscience, San Jose, CA, USA). The flow gating strategy and representative plots are shown in **Supplementary Figure 6**. BMDM were defined as CD11b^+^F4/80^+^. The intracellular markers (TNFα, IL-1β and iNOS) were evaluated from the macrophage population (CD11b^+^F4/80^+^).

### 2.3 Flow cytometry staining and cell analysis

The prepared single-cell suspensions from various tissues were stained with fluorescence-conjugated antibodies. Dead cells were stained using LIVE/DEAD™ Fixable Aqua Dead Cell Stain Kit (Thermofisher, USA) for 30 mins, followed by BD Fc block™ (CD16/32 clone, BD Bioscience, 553141) for 10 mins. The cell surface staining antibodies were resuspended in FACS buffer (1xPBS and 2% FBS) and incubated for 30 mins at 4°C followed by 2% fixation buffer in PBS (Thermofisher, J19943.K2) for 20 mins at 4°C. The cells were permeabilized by BD Perm/Wash Buffer (BD Bioscience, 554723) for 20 mins at 4°C followed by incubation with intracellular staining antibodies which were resuspended in Perm/Wash Buffer (BD, USA). The cells were then washed twice with the Perm/Wash Buffer prior to analysis.

The fluorescence intensity of the cells was detected by flow cytometers, MoFlo Astrios EQ (Beckman Coulter Life Sciences, Indianapolis, IN, USA), BD LSR Fortessa™ X-20 (BD Bioscience, San Jose, CA, USA) and/or Cytek® Aurora (Cytek®, Fremont, CA, USA). Unstained cells and fluorescence minus one (FMO) controls were used for compensation in the gating strategies. Data were analyzed using FlowJo software v10 (Tree Star Inc., Ashland, OR, USA).

### 2.4 Statistics

Statistical analysis was performed using GraphPad Prism 8.0.1 (GraphPad Software, La Jolla, CA, USA). Unpaired Student’s t-test was used. The data are presented as mean ± SEM. The statistical significance was considered as * *p* < 0.05; ** *p* < 0.01; *** *p* < 0.001.

## 3 Results

### 3.1 Innate immune cells reprogram in tissue-specific manner in aging

To study immune reprogramming in specific tissue microenvironments in aging, we studied innate immune cells in peripheral blood, peritoneal fluid, epididymal fat, liver, and colon in young and aged mice.

#### 3.1.1 Circulating monocytes are increased in peripheral blood of aged mice

**Figure 1:**
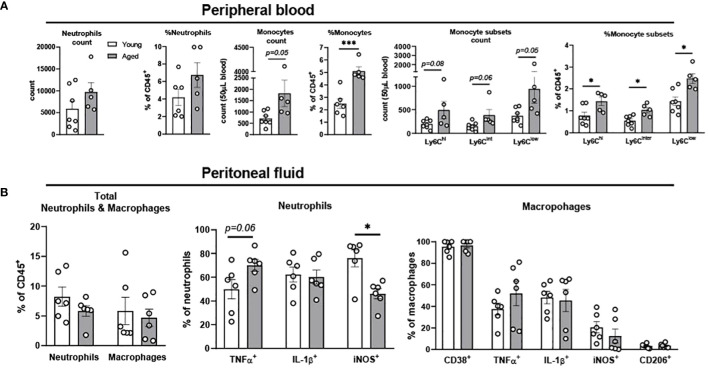
Characterization of neutrophils, monocytes, and macrophages in peripheral blood and peritoneal fluid of young and aged mice. Peripheral blood and peritoneal fluid from young (5-to-7-month-old, n=5-7) and aged (17-to-27-month-old, n=5-8) mice were subjected to flow cytometry for cell surface markers and cytokine expression analysis. **(A)** Peripheral blood neutrophils, monocytes, and monocyte subsets. The count data is measured in 50 uL blood. **(B)** Peritoneal fluid: Total neutrophils and macrophages (left), neutrophils (middle), and macrophages (right). Unpaired Student’s t-test was used. * p<0.05, *** p<0.001 aged vs. young.

Monocytes in circulation migrate to sites of inflammation and then differentiate into macrophages and dendritic cells to refill the pool of tissue immune cells (26). Circulating monocytes in mice are categorized into three subsets based on the surface expression of Ly6C; Ly6C^hi^, Ly6C^inter^ and Ly6C^low^ (27). Ly6C^hi^ cells are pro-inflammatory monocytes which rapidly migrate into inflamed tissue driven by the expression of CC-chemokine receptor 2 (CCR2). Ly6C^inter^ cells are pro-inflammatory monocytes which are differentiated from Ly6C^hi^. Ly6C^low^ subsets express low levels of CCR2, patrolling the vascular endothelium and engaging in tissue repair (27). To investigate neutrophils and monocytes in the circulation, peripheral blood of young and aged mice was investigated using flow cytometry. Aging did not change total neutrophil count and percentage, but significantly increased monocyte percentage in peripheral blood (**Figure 1A**). As expected, aging increased all three subsets of monocytes, pro-inflammatory Ly6C^hi^, Ly6C^inter^, and patrolling Ly6C^low^ compared to peripheral blood from young mice (**Figure 1A**). In summary, both pro-inflammatory and patrolling monocytes were increased in aging peripheral blood.

#### 3.1.2 Peritoneal fluid of aged mice exhibited mild trend of change in neutrophils and macrophages

The peritoneal fluid has abundant immune cells, predominantly with mature macrophages and neutrophils, and plays an important role in modulating immune response of the host (28–30). The total neutrophil percentage was not changed with aging, but the percentage of TNFα^+^ neutrophils showed a trend of increase in the aged mice (**Figure 1B**). While IL-1β^+^ neutrophils remained unchanged, iNOS^+^ neutrophils significantly decreased in aged mice (**Figure 1B**). TNFα^+^ macrophages showed a trend of increase, whereas macrophages with other pro-inflammatory markers of CD38, IL-1β, and iNOS did not differ between young and aged mice (**Figure 1B**). The anti-inflammatory CD206^+^ macrophages also did not change with age (**Figure 1B**). Collectively, the aging effect was quite mild in neutrophils and macrophages of peritoneal fluid.

#### 3.1.3 Pro-inflammatory myeloid cells are increased in epididymal fat of aged mice

**Figure 2:**
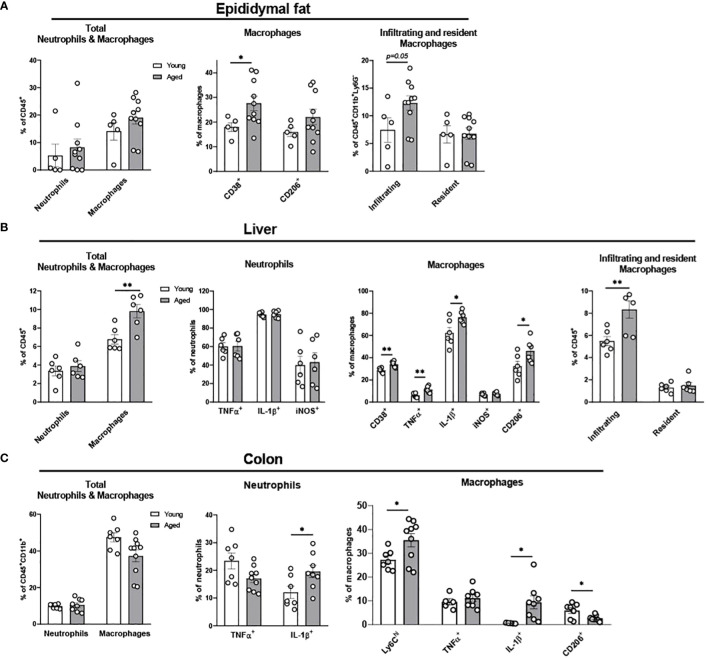
Characterization of myeloid population in epididymal fat, liver, and colon of young and aged mice. Young (5-to-7-month-old, n=5-7) and aged (17-to-27-month-old, n=5-8) mice were subjected to flow cytometry assessment of innate immune cells in epididymal fat, liver and colon. **(A)** Epididymal fat: Total neutrophils and macrophages (left), characteristics of macrophages (middle) and infiltrating/resident macrophages (right). **(B)** Liver: Total neutrophils and macrophages (far left), neutrophils (left), macrophages (right), and infiltrating/resident macrophages (far right). **(C)** Colonic total neutrophils and macrophages (left), and characteristics of colonic neutrophils (middle) and macrophages (right). Unpaired Student’s t-test was used. * p<0.05, ** p<0.01 aged vs. young.

We previously showed that aging increases adiposity and inflammation in adipose tissues, which further exacerbates insulin resistance in aging (31, 32). To further elucidate whether aging re-programs innate immune cells of neutrophils, monocytes, and macrophages in epididymal fat, we studied stromal vascular fraction (SVF) of epididymal fat in young and aged mice. While aging did not alter the percentage of total neutrophils and macrophages, the percentage of infiltrating macrophages showed a trend of increase in aging (**Figure 2A**). In addition, CD38^+^ pro-inflammatory macrophages were significantly elevated in aging. While the percentage of CD206^+^ anti-inflammatory macrophages also showed a trend of increase in aging, that trend was highly varied in aged mice (**Figure 2A**). Collectively, aging increased pro-inflammatory myeloid cells, mainly by increasing infiltrating macrophages and CD38^+^ pro-inflammatory macrophages in aging epididymal fat.

#### 3.1.4 The infiltration of pro-inflammatory macrophages is enhanced in the liver of aged mice

Fatty liver diseases, including non-alcoholic fatty liver disease (NAFLD) and nonalcoholic steatohepatitis (NASH), are more prevalent in aging (33–35). Here we examined whether innate immune cells in liver are altered in aging. Aging did not change the total percentage of neutrophils and pro-inflammatory TNFα^+^, IL-1β^+^, and iNOS^+^ neutrophils (**Figure 2B**). However, aging significantly elevated the total percentage of macrophages, and the pro-inflammatory CD38^+^, TNFα^+^, and IL-1β^+^ macrophages were significantly higher in the aging liver. Notably, although the infiltrating macrophages were significantly increased in aging liver, the resident macrophages (Kupffer cells) did not change (**Figure 2B**). Collectively, aging primarily affects macrophages, exhibiting the infiltration of pro-inflammatory macrophages in the liver.

#### 3.1.5 Myeloid cells in colon undergo pro-inflammatory programming in aging

The gastrointestinal tract has the largest pool of immune cells in the body due to the greatest amount of antigen exposure from diet and microbiome (36). To investigate if innate immune populations are altered in the aging colon, we used flow cytometry to assess innate immune cells of young and aged mice. The percentage of the total colonic neutrophils was similar between young and aged mice. However, aging significantly increased IL-1β^+^ pro-inflammatory neutrophils in colon compared to young mice colon, whereas aging did not significantly alter TNFα^+^ neutrophils (**Figure 2C**). Macrophage total percentage did not change in the aging colon (**Figure 2C**). However, a robust change of macrophage subpopulations was detected. Ly6C^hi^ infiltrating monocytes and macrophages were significantly increased in aging (**Figure 2C**). Also, IL-1β^+^ pro-inflammatory macrophages were significantly elevated in aged mice colon, whereas TNFα^+^ macrophages remained similar in aging (**Figure 2C**). Furthermore, aging colon showed significantly decreased CD206^+^ anti-inflammatory macrophages (**Figure 2C**). Collectively, aging significantly enhances pro-inflammatory neutrophils and macrophages, and reduces anti-inflammatory macrophages in the colon.

### 3.2 Bone marrow-derived macrophages from aged mice show impaired polarization in response to pro-inflammatory LPS stimulation

**Figure 3:**
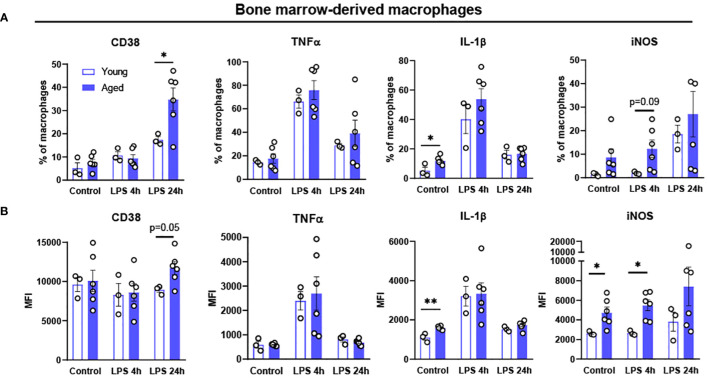
Polarization potential of bone-marrow derived macrophages (BMDM) from young and aged mice. Bone-marrow from young (5-month-old, n=3) and aged (17-to-18-month-old, n=6) mice were differentiated to BMDM by 10 ng/mL M-CSF for 7 days and then treated with 100 ng/mL LPS for 4 or 24 hours to promote pro-inflammatory polarization. Flow cytometry analysis of macrophages: **(A)** % of CD38,TNFα, IL-1β and iNOS; **(B)** MFI of CD38, TNFα, IL-1β and iNOS. Unpaired Student’s t-test was used. * p<0.05, ** p<0.01 aged vs. young.

Chronic exposure of pro-inflammatory cytokines in aging results in defective ability of BMDM to clear pathogens, suggesting aging driven functional alteration of BMDM (37, 38). To determine whether aging itself affects the inflammatory response of BMDM, BMDM from young (4-month-old) and aged (17-to-27-month-old) mice were treated with LPS 100 ng/mL for either 4 or 24 hours, and then pro-inflammatory markers were tested using flow cytometry. In BMDM from both young and aged mice, the expression of pro-inflammatory marker CD38 was expressed the most at 24 hours after LPS treatment (**Figure 3**). The BMDM from aged mice showed significantly increased CD38^+^ expression in 24 hours of LPS treatment. The expressions of the pro-inflammatory cytokines of TNFα and IL-1β peaked at 4 hours after LPS treatment, whereas iNOS expression was increased at 24 hours after LPS treatment (**Figure 3**). Interestingly, BMDM from aged mice had significantly elevated baseline expression of IL-1β compared to BMDM from young mice (**Figure 3**). Also, BMDM from aged mice showed significantly higher expression of iNOS at baseline and at 4 hours post-LPS treatment (**Figure 3**). Collectively, BMDM from aged mice exhibited higher expression of pro-inflammatory markers (CD38, IL-1β, and iNOS) compared to those in BMDM from young mice.

### 3.3 Aging innate immune cells show cell- and tissue-specific response to LPS-induced inflammation

Overwhelming evidences support that the elderly are more susceptible to infectious disease due to immune decline (39, 40). Although many vaccines are developed to combat life-threatening infections, the vaccine efficacy is lower in the elderly (41, 42) due to impaired immunity in the elderly. Our BMDM data above revealed that macrophages, which are key innate immune cells, have altered polarization in aging. To further test the differential response of aging innate immunity and its characteristic changes across the peripheral tissues in LPS-induced inflammation, low-dose LPS (0.75 mg/kg) was *i.p*. injected into young and aged mice, and tissues were collected after 6 hours. The dosage and the time of LPS treatment were chosen based on our preliminary titration study (data not shown). Considering that the elderly are more susceptible to infection (9), low-dose LPS injection was used in mice to induce acute inflammation to mimic the condition of the elderly when exposed to infection. Below, we have analyzed innate responses of the peripheral tissues in young and aged mice in response to the low dose LPS exposure.

#### 3.3.1 Myeloid cells in peripheral blood of aged mice exhibit a robust response to LPS

**Figure 4:**
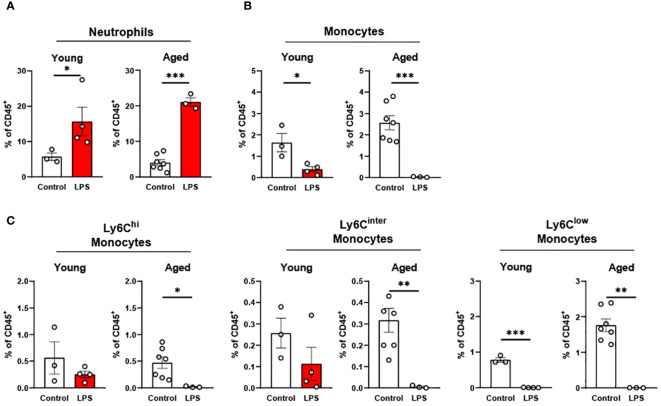
The response of peripheral blood immune cells to LPS in young and aged mice. Young (5-to-7-month-old, n=3-4) and aged (15-month-old, n=3-7) mice were subjected to either vehicle or LPS (*i.p.* 0.75 mg/kg) for 6 hours and the neutrophils and monocytes in peripheral blood were tested by flow cytometry. **(A)** Neutrophils **(B)** Monocytes **(C)** Monocyte subsets. Unpaired Student’s t-test was used. * p<0.05, ** p<0.01, *** p<0.001 LPS vs. control.

In peripheral blood, young mice showed significantly increased neutrophils upon LPS exposure (**Figure 4A**). Aged mice also exhibited increased neutrophils upon LPS treatment, whereas the response was more robust than in young mice (**Figure 4A**). Interestingly, total monocyte percentage was significantly reduced in both young and old mice in response to LPS (**Figure 4B**). Furthermore, the monocyte subsets of Ly6C^hi^, Ly6C^inter^ and Ly6C^low^ were all decreased by LPS in both young and aged mice (**Figure 4C**). Of note, old mice showed a more pronounced reduction of Ly6C^hi^, Ly6C^inter^ and Ly6C^low^ monocytes as compared to that of young mice (**Figure 4C**). Collectively, peripheral blood neutrophils were increased, but total monocytes and monocyte subsets were decreased in both young and aged mice. On the other hand, aged mice showed more robust change of neutrophils and monocyte subsets in response to LPS as compared to young mice.

#### 3.3.2 The responses of peritoneal neutrophils and macrophages to LPS are impaired in aging

**Figure 5:**
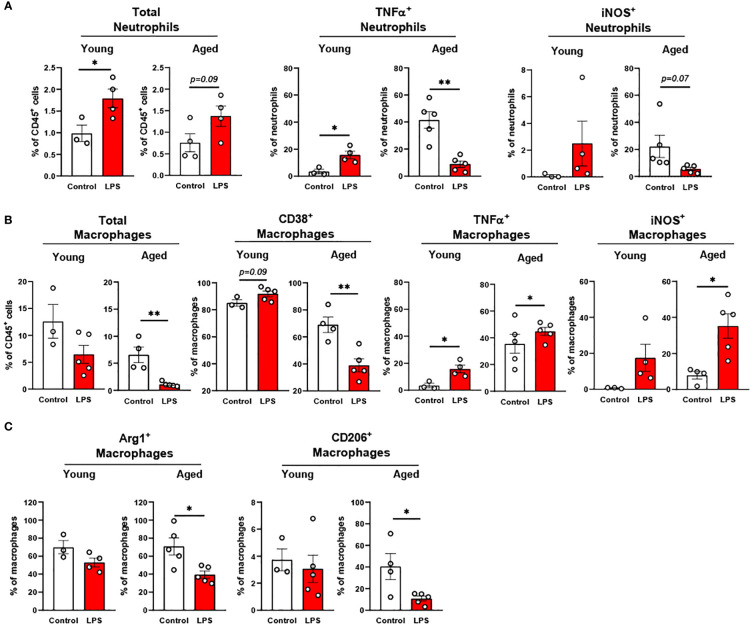
The response of peritoneal innate immune cells to LPS in young and aged mice. Young (5-to-7-month-old, n=3-4) and aged (18-to-24-month-old, n=3-5) mice were subjected to either vehicle or LPS (*i.p.* 0.75 mg/kg) for 6 hours, and the neutrophils and macrophages were assessed by flow cytometry. **(A)** Total neutrophils, TNFα^+^ or iNOS^+^ neutrophils; **(B)** Total macrophages, CD38^+^, TNFα^+^ or iNOS^+^ macrophages; **(C)** Arg1^+^ or CD206^+^ macrophages. Unpaired Student’s t-test was used. * p<0.05, ** p<0.01 LPS vs. control.

In the peritoneal fluid, young mice showed significantly elevated neutrophil percentage in response to LPS. The aged mice also showed a trend of increase in neutrophil percentage by LPS treatment (**Figure 5A**). Interestingly, the secretion of pro-inflammatory cytokines in neutrophils was the opposite between young and aged mice in response to LPS (**Figure 5A**): In young mice, TNFα^+^ and iNOS^+^ neutrophils were increased in peritoneal fluid; counterintuitively, aged mice exhibited decreased TNFα^+^ and iNOS^+^ neutrophils upon LPS exposure. Next, we found that the young mice displayed a trend of decrease in total macrophage percentage in response to LPS, whereas aged mice exhibited a significantly lower macrophage percentage under LPS treatment (**Figure 5B**). On the other hand, TNFα and iNOS expressing pro-inflammatory macrophages were significantly increased in both young and aged mice (**Figure 5B**). Of note, aged mice showed a less robust increase of TNFα^+^ macrophages than did young mice (**Figure 5B**). On the contrary, aged mice had a more significant increase of iNOS^+^ macrophages compared to young mice under LPS (**Figure 5B**). The response of pro-inflammatory CD38^+^ macrophages in LPS was also different in young and aged mice. Young mice showed increased CD38^+^ pro-inflammatory macrophages in response to LPS, while aged mice showed significantly decreased CD38^+^ macrophages (**Figure 5B**). In addition to pro-inflammatory markers, anti-inflammatory markers Arg1 and CD206 were also tested in macrophages. Under LPS exposure, young mice showed merely a trend of decreased Arg1^+^ macrophages, whereas aged mice showed significantly decreased Arg1^+^ macrophages (**Figure 5C**). Similarly, young mice did not show an altered CD206^+^ macrophage percentage, whereas aged mice showed a significantly reduced CD206^+^ macrophage percentage (**Figure 5C**). Collectively, aged mice showed an impaired response of neutrophils and macrophages to LPS. The macrophage subsets in aged mice showed differential responses to LPS depending on cytokine production.

#### 3.3.3 Aging increases the responsiveness of adipose macrophages to LPS stimulation

**Figure 6:**
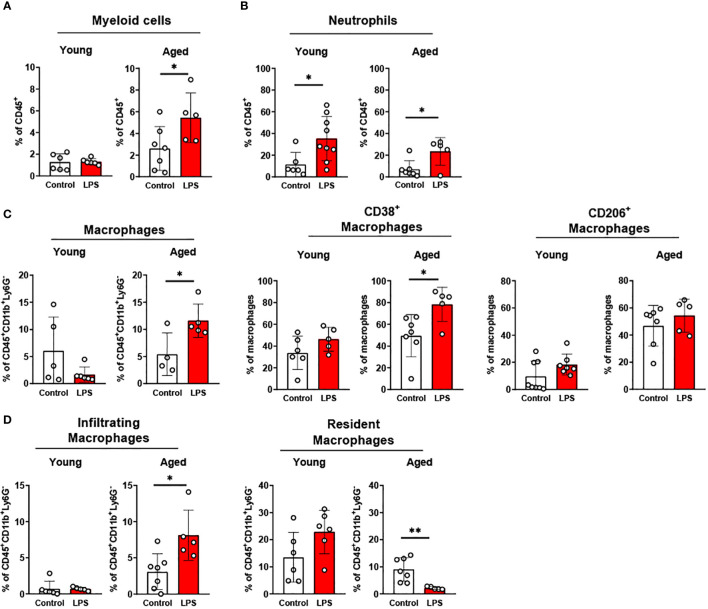
The response of adipose innate immune cells to LPS in young and aged mice. Young (5-to-7-month-old, n=5-6) and aged (18-to-24-month-old, n=4-7) mice were subjected to either vehicle or LPS (*i.p.* 0.75 mg/kg) for 6 hours, and the innate immune cells from SVF of epididymal fat were used for flow cytometry analysis. **(A)** Myeloid cells **(B)** Neutrophils **(C)** Macrophages, as well as CD38^+^ or CD206 ^+^ macrophage subset, and **(D)** Infiltrating and resident macrophages. Unpaired Student’s t-test was used. * p<0.05, ** p<0.01 LPS vs. control.

In epididymal fat of young mice, LPS treatment did not change total myeloid cells whereas aged mice showed significantly increased myeloid cells (**Figure 6A**). The percentage of neutrophils was significantly increased following LPS stimulation in both groups (**Figure 6B**); however, the response was less pronounced in the aged mice compared to young mice (**Figure 6B**). On the other hand, macrophages in aged epididymal fat were more responsive to LPS than macrophages in epididymal fat from young mice. Total macrophages in young mice were not altered under LPS exposure, whereas the total macrophages in aged mice exhibited a significantly increased in epididymal fat (**Figure 6C**). Similarly, the macrophage subsets showed significant changes in aged mice but not in young mice (**Figure 6C**): While young mice did not show an altered macrophage subpopulation in response to LPS, aged mice revealed significantly increased CD38^+^ pro-inflammatory macrophages. The CD206^+^ anti-inflammatory macrophage population was not changed in either young or aged mice in response to LPS treatment (**Figure 6C**). Furthermore, in aged mice, there was significantly elevated macrophage infiltration and reduced resident macrophages upon LPS treatment (**Figure 6D**). Collectively, aging decreased neutrophil response but increased macrophage responses to inflammatory stimuli in epididymal fat.

#### 3.3.4 Liver macrophages are more responsive to LPS in aged mice

**Figure 7:**
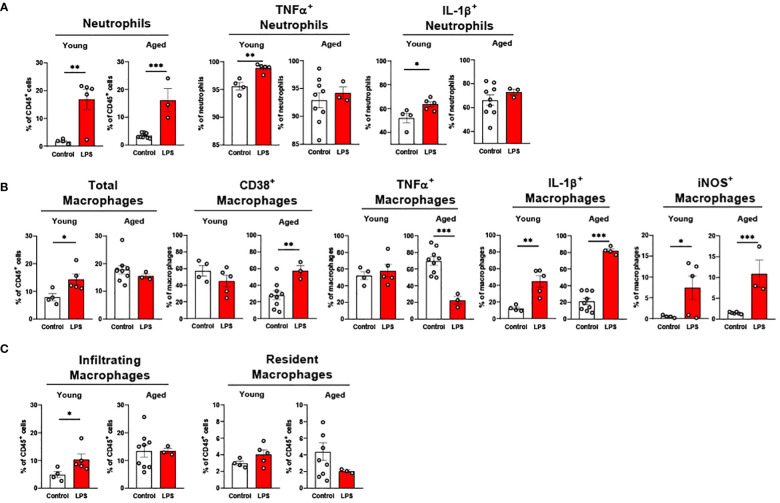
The response of liver innate immune cells to LPS in young and aged mice. Young (5-to-7-month-old, n=4-5) and aged (20-month-old, n=3) mice were subjected to either vehicle or LPS (*i.p.* 0.75 mg/kg) for 6 hours, and the neutrophils and macrophages were assessed by flow cytometry. **(A)** Total neutrophils, TNFα^+^ or IL-1β^+^ neutrophils; **(B)** Total macrophages, CD38^+^, TNFα^+^, IL-1β^+^ or iNOS^+^ macrophages; **(C)** Infiltrating and resident macrophages. Unpaired Student’s t-test was used. * p<0.05, ** p<0.01, *** p<0.001 LPS vs. control.

Liver neutrophils were significantly elevated in both young and old mice in response to LPS (**Figure 7A**). Young mice showed significantly increased TNFα^+^ and IL-1β^+^ neutrophils in response to LPS, whereas these cells in livers of aged mice did not respond to LPS (**Figure 7A**). Old mice presented a robust change of pro-inflammatory macrophages compared to young mice in response to LPS (**Figure 7B**). CD38^+^ macrophages in young mice did not change, whereas old mice showed significantly elevated CD38^+^ macrophages upon LPS challenge (**Figure 7B**). In young mice, TNFα^+^ macrophages did not respond to LPS; however, this cell type was significantly reduced in old mice (**Figure 7B**). The IL-1β^+^ and iNOS^+^ macrophages were significantly increased in both young and old mice, but aged mice showed a more robust increase in those macrophages (**Figure 7B**). Interestingly, young mice showed increased infiltrating macrophages in response to LPS exposure, whereas aged mice did not show a change in macrophage infiltration (**Figure 7C**). The resident macrophages were not significantly altered in both young and aged mice following LPS treatment (**Figure 7C**). Collectively, liver neutrophils responded similarly between young and old mice, whereas pro-inflammatory macrophages from the livers of aged mice showed a more potent response to LPS.

#### 3.3.5 Colonic macrophages have dampened response to LPS

**Figure 8:**
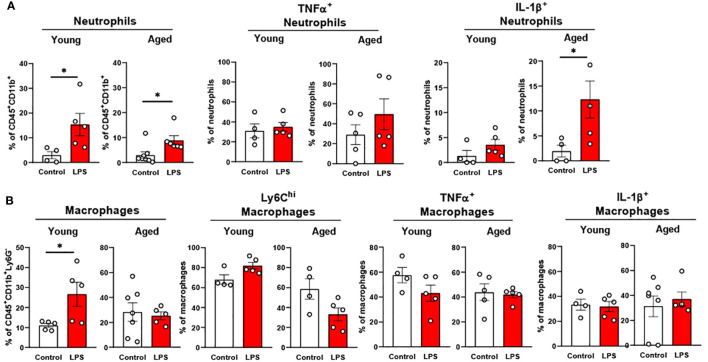
The response of colonic neutrophils and macrophages to LPS in young and aged mice. Young (5-to-7-month-old, n=4-5) and aged (18-to-24-month-old, n=5-7) mice were subjected to either vehicle or LPS (*i.p.* 0.75 mg/kg) for 6 hours, and the neutrophils and macrophages were assessed by flow cytometry. **(A)** Total neutrophils, TNFα^+^ or IL-1β^+^ neutrophils; **(B)** Total macrophages, Ly6C^hi^ infiltrating macrophages, TNFα^+^ or IL-1β^+^ macrophages. Unpaired Student’s t-test was used. * p<0.05 LPS vs. control.

Our data above support a significant aging effect of increased pro-inflammatory innate immune cells in the colon. Interestingly, colonic innate immune cells from both young and aged mice displayed relatively mild responsiveness to LPS challenge. Total neutrophil percentage was significantly higher in both young and aged mice colons, but aged mice colons showed a smaller increase of neutrophils in response to LPS as compared to young mice (**Figure 8A**). TNFα^+^ neutrophils were steady in both young and aged mice upon LPS (**Figure 8A**). While in young mice, IL-1β^+^ neutrophils remained non-responsive to LPS, aged mice showed significantly escalated IL-1β^+^ neutrophils in LPS exposure (**Figure 8A**). Total macrophage population from young mouse colons was increased by LPS, whereas the percentage of the total macrophages in aged mouse colons did not change (**Figure 8B**). Also, young mice showed a trend of increased Ly6C^hi^ macrophages, whereas aged mice showed a trend of decreased Ly6C^hi^ macrophages; however, neither reached statistical significance (**Figure 8B**). LPS did not alter TNFα^+^ and IL-1β^+^ macrophages in both young and aged mice (**Figure 8B**). Overall, total neutrophils and macrophages increased in response to LPS. While pro-inflammatory neutrophils were increased significantly with LPS stimulation in aged mice, pro-inflammatory macrophages barely responded to LPS in either young or aged colons.

**Figure 9:**
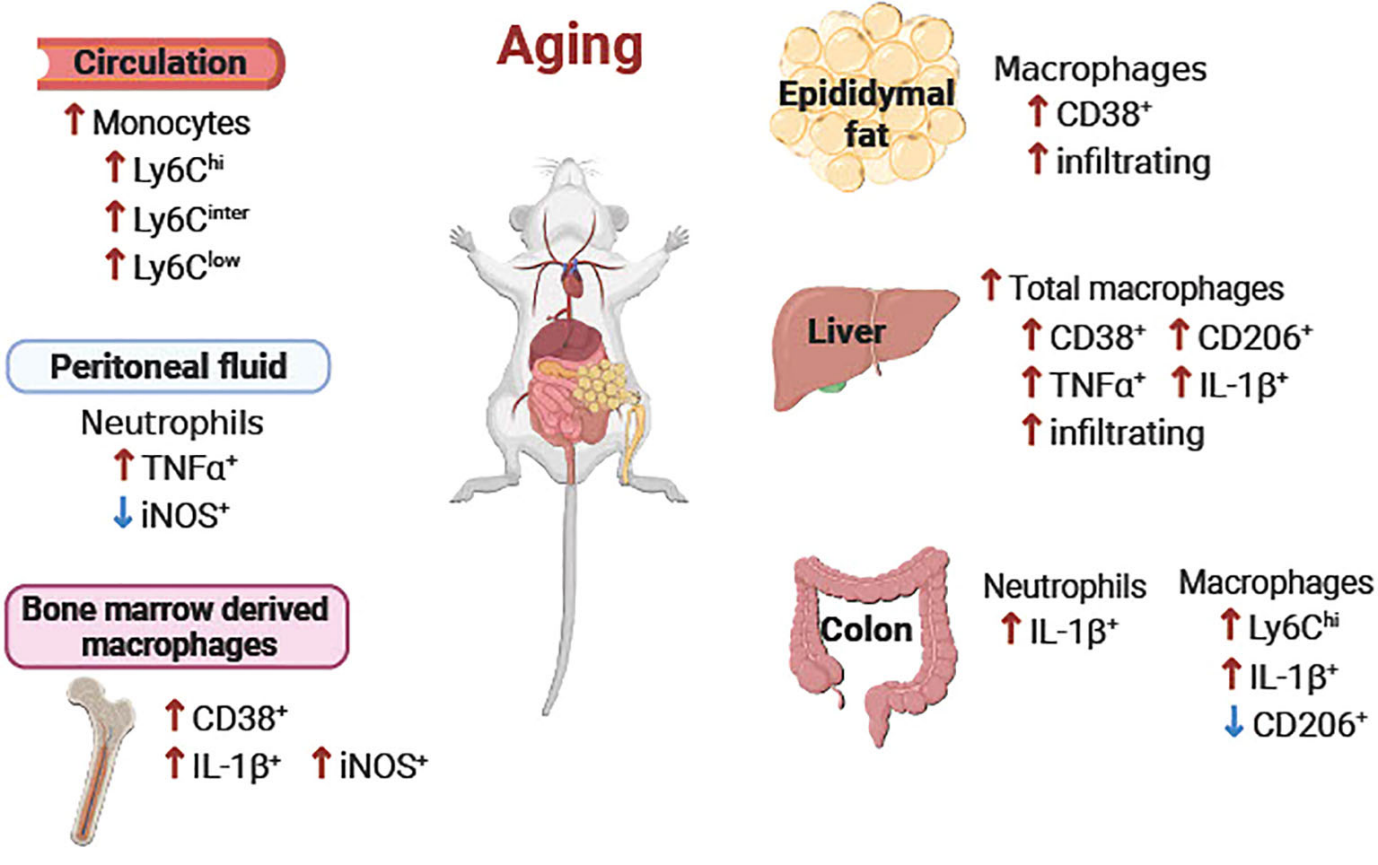
Schematic diagram of the age-associated alterations of immune cells in peripheral tissues. Overall, pro-inflammatory neutrophils, monocytes and macrophages were increased in aging in peripheral blood, peritoneal fluid, epididymal fat, liver, colon, and bone marrow-derived macrophages. ↑ or ↓ aged vs. young.

The collective data of the age-associated alterations of immune cells in each tissue are summarized in **Figure 9**. Also, responses of the immune cells to LPS in each tissue are summarized in **Table 1**.

**Table 1:** Summary of aging under LPS.

	Neutrophils			Monocytes/Macrophages		
	Population	Young	Aged	Population	Young	Aged
**Peripheral blood**	Total	↑	↑↑↑	Total Ly6C^hi^ Ly6C^inter^ Ly6C^low^	↓ - - ↓↓↓	↓↓↓ ↓ ↓↓ ↓↓
**Peritoneal fluid**	Total TNFα iNOS	↑ ↑ -	- ↓↓ -	Total CD38 TNFα iNOS Arg1 CD206	- - ↑ - - -	↓↓ ↓↓ ↑ ↑ ↓ ↓
**Epididymal fat**	Total	↑	↑	Total CD38 CD206 Infiltrating Resident	- - - - -	↑ ↑ - ↑ ↓↓
**Liver**	Total TNFα IL-1β	↑↑ ↑↑ ↑	↑↑↑ - -	Total CD38 TNFα IL-1β iNOS Infiltrating Resident	↑ - - ↑↑ ↑ ↑ -	- ↑↑ ↓↓↓ ↑↑↑ ↑↑↑ - -
**Colon**	Total TNFα IL-1β	↑ - -	↑ - ↑	Total Ly6C^hi^ TNFα IL-1β	↑ - - -	- - - -

↑ or ↓ *p*<0.05, ↑↑ or ↓↓ *p*<0.01, ↑↑↑ or ↓↓↓ *p*<0.001 LPS vs. control.

## 4 Discussion

Emerging evidence highlights the importance of innate immunity in aging and age-associated diseases (2, 4, 12, 43). We studied innate immune programming of a wide range of peripheral tissues under both normal aging and an LPS-induced inflammatory state. Our current study reveals that aging impairs innate immunity in peripheral tissues in both normal and endotoxic conditions. Interestingly we found that the activation/response of the innate immune cells upon inflammatory stimulus is distinctively tissue/cell-dependent in aging.

Our data from peripheral blood showed that pro-inflammatory Ly6C^hi^ monocytes were significantly increased in aging (**Figure 1A**). The data are in agreement with previous studies showing higher monocytes in older adults compared to young adults (44, 45), and in further support of previous findings that aging is associated with chronic systemic inflammation (2, 3, 37). Under steady state in aging, we observed that colons from the aged mice exhibited enhanced infiltration of macrophages, and pro-inflammatory activation of neutrophils and macrophages (**Figure 2C**), which could contribute to systemic inflammation during aging. It is known that colon is chronically exposed to inflammatory stimuli due to microbial dysbiosis and increased gut permeability during aging (37, 46–50). The chronic exposure to inflammatory stimuli reduces phagocytic activity of macrophages in removing pathogens (37), which would perpetuate colonic inflammation. The age-associated colonic inflammation likely further contributes to increased vulnerability to inflammatory bowel disease in aging as we and others have reported (51–53). Collectively, our data revealed pro-inflammatory activation of colonic neutrophils and macrophages, contributing to systemic inflammation in aging.

Aging is associated with obesity, a condition influenced by dietary and lifestyle choices which significantly contribute to the prevalence of insulin resistance and metabolic diseases in the elderly (54, 55). Epididymal fat and liver are major metabolic tissues extensively studied in chronic inflammatory diseases in the context of inflammation and insulin resistance (56–59). We demonstrated that macrophages in epididymal fat and liver are responsive to age-associated inflammation, exhibiting increased infiltration of macrophages (**Figure 2A, B**). This is in line with previous reports by us and others showing accumulation of monocytes and macrophages in adipose tissue and liver in aging (31, 60). It has been reported that accumulation of senescence cell-driven mediators (SASP) in aging impairs leukocyte trafficking, thus exhibiting dysregulated adhesion/chemokine mediator expressions in aged endothelium, immune cells in the circulation, and the tissue microenvironment (61–63). We observed increased CD38^+^ pro-inflammatory macrophages in epididymal fat and liver in aging (**Figure 2A, B**), which likely is due to increased SASP-driven CD38 expression in macrophages (64). Though it is not clear if macrophage senescence itself could directly increase level of CD38, the senescence of macrophages is known to induce defective insulin signaling in human adipocytes (65). While ample evidence supports increased infiltration of macrophages into adipose tissue in aging, the change of resident macrophages in adipose tissue has been debatable. In our study, resident macrophages (CD45^+^CD11b^+^F4/80^low^) were not changed in aging (**Figure 2A**). As another study reported increased resident macrophages in adipose tissue (F4/80^+^CD11b^+^CD11c^-^) (64), these discrepancies suggest a heterogeneity of resident macrophages. Assessment of functional differences between subpopulations within resident macrophages using single-cell RNA sequencing would be beneficial. Interestingly, we found that CD206^+^ anti-inflammatory macrophages were also increased in aging liver (**Figure 2B**); This is in line with the report showing counteract response of the macrophages to adapt to age-associated pro-inflammatory state (66). Recent study unraveled a novel mechanism of the gut-liver axis, suggesting that commensal microbe-derived components lead to suppression of liver-derived molecules which in turn regulate proliferation of intestinal stem cells (67). Investigating this mechanism in the context of aging would be interesting in order to determine whether there is a possible link between tissue crosstalk, immune cell activation, and age-associated inflammation. Collectively, our data suggest that alteration of the tissue microenvironment in aging underlies the pro-inflammatory activation of macrophages in epididymal fat and liver in aging, thus contributing to systemic inflammation.

Given that impaired immune responses to vaccines and infection leads to high mortality in the elderly, we further studied how the innate immune cells respond to systemic inflammation in peripheral tissues in young and aged mice. The myeloid cell populations in tissue consist of resident immune cells and the recruited infiltrating immune cells from the circulation. In our study, we observed that the aged mice showed a greater increase of blood neutrophils in LPS exposure as compared to young mice (**Figure 4A**). On the other hand, we found that the neutrophil expansion in peripheral tissues (peritoneal fluid, epididymal fat, and colon) was less robust in aged mice than young mice with LPS treatment (**Figures 5A**, **6B**, **8A**). Our data are in line with the reports of low chemotaxis of neutrophils in the elderly (68, 69). Together, our data suggest that aging delays neutrophil recruitment into tissues, in response to an inflammatory insult.

Compared to neutrophils, infiltrating monocytes/macrophages showed distinctive profiles in response to LPS. We found that total circulating monocytes and their subsets were all significantly reduced in response to LPS in both young and aged mice, but aged mice showed a more significant reduction compared to young mice (**Figure 4C**). This result is likely attributed to the higher baseline number of circulating monocytes in aged mice in a steady state (**Figure 1A**). Our data is consistent with other reports that endotoxin-induced inflammation decreases the number of monocytes in humans in the early stages of inflammation (70–73). In contrast, another study reported that immature monocytes are promptly released to circulation in response to bacterial and viral infection (72, 74, 75). While emergency monopoiesis can enhance production of monocytes in the bone marrow under inflammatory stress (76), it replenishes the monocyte population within 4-8 hours after endotoxin exposure (72). We used low-dose LPS for 6 hours in our experiment, which is in line with the report above. We observed the differential expansion of monocytes/macrophages within tissues in aging under LPS (**Figures 5B**, **6D**, **7C**, **8B**). Collectively, under LPS, monocytes in circulation exhibited a significant reduction whereas the tissue monocytes/macrophage were differentially regulated in young and aged mice.

Our data demonstrated that the inflammatory response of innate immune cells is cell- and/or tissue-dependent. We observed that young mice showed enhanced pro-inflammatory macrophages in peritoneal fluid and liver upon LPS exposure (**Figures 5B**, **7B**). Our results are in line with the report showing that the peritoneal macrophages can rapidly infiltrate liver through non-vascular routes within 2 hours of post-injury (77). On the contrary, we observed the immune cells in epididymal fat from young mice were barely responsive to LPS (**Figure 6C**), suggesting an immune tolerance specific to the tissue niche under an inflammatory state. Interestingly, depending on tissue/cell type, we found differential responses to LPS challenge in immune cells in aged mice, showing either hyper-reactivity, hypo-reactivity, or desensitization/tolerance (**Figures 4B**, **6C**, **7A, 7B**). Accumulating evidences indicate a correlation between inflammaging and trained immunity which is the response of immune cells becoming higher and quicker upon subsequent inflammatory exposure (78, 79). On the other hand, others have reported reduced responsiveness or tolerance of macrophages, exhibiting downregulated toll-like receptor 4 in consequential low-grade inflammatory challenge; this is a protective mechanism to limit cell damage and prevent septic shock (80–82). The macrophage response resulting in trained immunity or immune-tolerance could be determined by multiple factors such as dose/time of LPS, and/or tissue microenvironmental factors (77, 83, 84). Better understanding of cell-specific responses and factors determining immune responses in aging will be beneficial for future development of therapeutic approaches for inflammaging-related chronic disease in the elderly (85). Taken together, our study revealed that the response of immunity to LPS challenges in aged mice is impaired tissue- and cell-dependent.

Unlike other tissues, colonic macrophages with pro-inflammatory cytokine expressions were not altered in response to LPS (0.75 mg/kg) in young mice (**Figure 8B**). However, others have shown that a higher dose of LPS (2-5 mg/kg) induces colon inflammation (86, 87), indicating the response of the colon to exogenous LPS is dose-dependent. Of note, colonic immune cells responded to intrinsic sterile inflammation induced by aging, thus exhibiting significantly elevated pro-inflammatory profiles (**Figure 2C**). This suggests that colonic immune cells are likely more responsive to intrinsic inflammatory factors rather than extrinsic factors. Notably, colonic macrophages from aged mice also did not show response to LPS (**Figure 8B**). The tolerance of aged colonic cells to LPS could be due to macrophage dysfunction caused by chronic exposure to sterile inflammation in aging. Indeed, it has been reported that prolonged exposure to pro-inflammatory mediators suppresses the macrophage functions (37).

Sexual dimorphism is an important biological variable in immune response. Age-associated changes of immune cells show sex-dependent variability, reflected in cell types as well as the degree of immune responses (88); this potentially explains why males are more susceptible to infections while females are more vulnerable to autoimmune diseases (89–92). In chronic low-grade inflammation, peripheral blood monocytes in women exhibited higher immune activation transcriptomes compared to men (93). Leukocyte trafficking, especially of macrophages into peritoneal fluid, was higher in young female mice than young male mice, but it was similar in both old male and old female mice (61, 62). Even though the numbers of infiltrating macrophages are similar between sexes, a greater pro-inflammatory shift of macrophages was reported in male mice versus an anti-inflammatory shift in female mice was reported under infection and aging (94, 95). In the current long-term aging study, we focused on male mice. Future studies in females are needed to elucidate phenotype and functional change of innate immunity across various tissues during aging and under disease conditions.

Ample evidence shows that neutrophils, monocytes, and macrophages have essential roles in wound repair (96–98). These immune cells also shift their phenotype and functional properties during the repair stage. The previous report showed that the mature macrophages infiltrated from the peritoneum to the local site of injury can be alternatively activated 12 hours after local injury to secure the tissue integrity (99). Aging reduces the tissue regeneration ability after injury, which possibly can be attributed to age-associated impaired functions of immune cells (100, 101). The information is limited on what mechanisms of innate immune cells are involved in defective tissue repair in aging. While our data have shed light on aging innate immunity, it is important to note that in our study, the phenotypical and functional characteristics of myeloid cells were studied at 6 hours after LPS injection, a snapshot of the inflammatory state. Long-term longitudinal studies throughout aging are critically needed.

Age-associated chronic diseases, including obesity, type 2 diabetes, and atherosclerosis, are tightly associated with immune dysfunction (102). Current available therapeutic approaches such as anti-integrin/chemokine therapies, peptide inhibitors, and anti-aging drugs like metformin and rapamycin, are known to regulate leukocyte trafficking and block pro-inflammatory mediators in the treatment of inflammation-driven diseases (62). However, these treatments are often accompanied with various side effects (62, 103, 104) due to lack of cell specificity and/or unsatisfactory efficacy in older adults. The current knowledge in tissue-specific immune programming in aging is very limited, and the mediators responsive to the local tissue environment in aging are largely unexplored. Our study underscores the cell-specific and tissue-specific alteration of innate immunity in aging. We investigated the dynamics of neutrophils and macrophages in various peripheral tissues under normal aging as well as innate immune responses to inflammatory stimulus in aging. The insights gained from our study offer a crucial foundation for developing tissue-specific prevention and therapeutic strategies for age-related chronic diseases. Future studies to further elucidate cell-autonomous effects and the interaction among local and systemic mediators using single-cell RNA sequencing, spatial transcriptomics, and cell-specific gene targeting would help to obtain better understanding of immunoregulation in aging and advance the development of cell-and tissue-specific therapeutic opportunities.

## Data Availability

The original contributions presented in the study are included in the article/**Supplementary Material**. Further inquiries can be directed to the corresponding author.

## References

[1] Dorshkind K Montecino-Rodriguez E Signer RAJ. The ageing immune system: is it ever too old to become young again? Nat Rev Immunol. (2009) 9:57–62. doi: 10.1038/nri2471 19104499

[2] Kennedy BK Berger SL Brunet A Campisi J Cuervo AM Epel ES. Geroscience: linking aging to chronic disease. Cell. (2014) 159:709–13. doi: 10.1016/j.cell.2014.10.039 PMC4852871 25417146

[3] Franceschi C Garagnani P Parini P Giuliani C Santoro A. Inflammaging: A new immune-metabolic viewpoint for age-related diseases. Nat Rev Endocrinol. (2018) 14:576–90. doi: 10.1038/s41574-018-0059-4 30046148

[4] Frasca D Blomberg BB Paganelli R. Aging, obesity, and inflammatory age-related diseases. Front Immunol. (2017) 8:1745. doi: 10.3389/fimmu.2017.01745 29270179 PMC5725402

[5] Harris TB Ferrucci L Tracy RP Corti MC Wacholder S Ettinger WH. Associations of elevated interleukin-6 and C-reactive protein levels with mortality in the elderly∗∗Access the “Journal club” Discussion of this paper at http:/www.Elsevier.Com/locate/ajmselect. Am J Med. (1999) 106:506–12. doi: 10.1016/S0002-9343(99)00066-2 10335721

[6] Bruunsgaard H Ladelund S Pedersen AN Schroll M Jørgensen T Pedersen BK. Predicting death from tumour necrosis factor-alpha and interleukin-6 in 80-year-old people. Clin Exp Immunol. (2003) 132:24–31. doi: 10.1046/j.1365-2249.2003.02137.x 12653832 PMC1808682

[7] Furman D Chang J Lartigue L Bolen CR Haddad F Gaudilliere B. Expression of specific inflammasome gene modules stratifies older individuals into two extreme clinical and immunological states. Nat Med. (2017) 23:174–84. doi: 10.1038/nm.4267 PMC5320935 28092664

[8] Giovannini S Onder G Liperoti R Russo A Carter C Capoluongo E. Interleukin-6, C-reactive protein, and tumor necrosis factor-alpha as predictors of mortality in frail, community-living elderly individuals. J Am Geriatr Soc. (2011) 59:1679–85. doi: 10.1111/j.1532-5415.2011.03570.x PMC4321727 21883115

[9] Collaborators GBDCoD. Global, regional, and national age-sex-specific mortality for 282 causes of death in 195 countries and territories, 1980-2017: A systematic analysis for the global burden of disease study 2017. Lancet. (2018) 392:1736–88. doi: 10.1016/S0140-6736(18)32203-7 PMC6227606 30496103

[10] Akbar AN Henson SM Lanna A. Senescence of T lymphocytes: implications for enhancing human immunity. Trends Immunol. (2016) 37:866–76. doi: 10.1016/j.it.2016.09.002 27720177

[11] Ma S Wang C Mao X Hao Y. B cell dysfunction associated with aging and autoimmune diseases. Front Immunol. (2019) 10:318. doi: 10.3389/fimmu.2019.00318 30873171 PMC6400972

[12] De Maeyer RPH Chambers ES. The impact of ageing on monocytes and macrophages. Immunol Lett. (2021) 230:1–10. doi: 10.1016/j.imlet.2020.12.003 33309673

[13] Gordon S Martinez FO. Alternative activation of macrophages: mechanism and functions. Immunity. (2010) 32:593–604. doi: 10.1016/j.immuni.2010.05.007 20510870

[14] Mantovani A Sica A Locati M. Macrophage polarization comes of age. Immunity. (2005) 23:344–6. doi: 10.1016/j.immuni.2005.10.001 16226499

[15] Italiani P Boraschi D. From monocytes to M1/M2 macrophages: phenotypical vs. functional differentiation. Front Immunol. (2014) 5:514. doi: 10.3389/fimmu.2014.00514 25368618 PMC4201108

[16] Atri C Guerfali F Laouini D. Role of human macrophage polarization in inflammation during infectious diseases. Int J Mol Sci. (2018) 19:1801. doi: 10.3390/ijms19061801 29921749 PMC6032107

[17] Kang B Alvarado LJ Kim T Lehmann ML Cho H He J. Commensal microbiota drive the functional diversification of colon macrophages. Mucosal Immunol. (2020) 13:216–29. doi: 10.1038/s41385-019-0228-3 PMC7039809 31772323

[18] Gordon S Plüddemann A. Tissue macrophages: heterogeneity and functions. BMC Biol. (2017) 15:53. doi: 10.1186/s12915-017-0392-4 28662662 PMC5492929

[19] Martinez FO Gordon S. The M1 and M2 paradigm of macrophage activation: time for reassessment. F1000Prime Rep. (2014) 6:13. doi: 10.12703/P6-13 24669294 PMC3944738

[20] Pence BD Yarbro JR. Aging impairs mitochondrial respiratory capacity in classical monocytes. Exp Gerontol. (2018) 108:112–7. doi: 10.1016/j.exger.2018.04.008 29655929

[21] Yarbro JR Pence BD. Classical monocytes from older adults maintain capacity for metabolic compensation during glucose deprivation and lipopolysaccharide stimulation. Mech Ageing Dev. (2019) 183:111146. doi: 10.1016/j.mad.2019.111146 31493436 PMC7027594

[22] Wagner AD Goronzy JJ Weyand CM. Functional profile of tissue-infiltrating and circulating cd68+ Cells in giant cell arteritis. Evidence for Two Components of the Disease. J Clin Invest. (1994) 94:1134–40. doi: 10.1172/JCI117428 PMC295180 8083354

[23] Ma X Lin L Yue J Pradhan G Qin G Minze LJ. Ghrelin receptor regulates hfcs-induced adipose inflammation and insulin resistance. Nutr Diabetes. (2013) 3:e99. doi: 10.1038/nutd.2013.41 24366371 PMC3877431

[24] Ma X Lin L Yue J Wu CS Guo CA Wang R. Suppression of ghrelin exacerbates hfcs-induced adiposity and insulin resistance. Int J Mol Sci. (2017) 18:1302. doi: 10.3390/ijms18061302 28629187 PMC5486123

[25] Weischenfeldt J Porse B. Bone marrow-derived macrophages (Bmm): isolation and applications. Cold Spring Harbor Protoc. (2008) 2008:pdb.prot5080. doi: 10.1101/pdb.prot5080 21356739

[26] Jakubzick CV Randolph GJ Henson PM. Monocyte differentiation and antigen-presenting functions. Nat Rev Immunol. (2017) 17:349–62. doi: 10.1038/nri.2017.28 28436425

[27] Yang J Zhang L Yu C Yang XF Wang H. Monocyte and macrophage differentiation: circulation inflammatory monocyte as biomarker for inflammatory diseases. biomark Res. (2014) 2:1. doi: 10.1186/2050-7771-2-1 24398220 PMC3892095

[28] Liu M Silva-Sanchez A Randall TD Meza-Perez S. Specialized immune responses in the peritoneal cavity and omentum. J Leukocyte Biol. (2021) 109:717–29. doi: 10.1002/JLB.5MIR0720-271RR PMC7921210 32881077

[29] Zou G Wang J Xu X Xu P Zhu L Yu Q. Cell subtypes and immune dysfunction in peritoneal fluid of endometriosis revealed by single-cell rna-sequencing. Cell Bioscience. (2021) 11:98. doi: 10.1186/s13578-021-00613-5 34039410 PMC8157653

[30] Buscher K Wang H Zhang X Striewski P Wirth B Saggu G. Protection from septic peritonitis by rapid neutrophil recruitment through omental high endothelial venules. Nat Commun. (2016) 7:10828. doi: 10.1038/ncomms10828 26940548 PMC4785224

[31] Lin L Lee JH Buras ED Yu K Wang R Smith CW. Ghrelin receptor regulates adipose tissue inflammation in aging. Aging. (2016) 8:178–91. doi: 10.18632/aging.v8i1 PMC4761721 26837433

[32] Lin L Saha PK Ma X Henshaw IO Shao L Chang BHJ. Ablation of ghrelin receptor reduces adiposity and improves insulin sensitivity during aging by regulating fat metabolism in white and brown adipose tissues. Aging Cell. (2011) 10:996–1010. doi: 10.1111/j.1474-9726.2011.00740.x 21895961 PMC3215833

[33] Wang Z Xu M Peng J Jiang L Hu Z Wang H. Prevalence and associated metabolic factors of fatty liver disease in the elderly. Exp Gerontol. (2013) 48:705–9. doi: 10.1016/j.exger.2013.05.059 23721951

[34] Koehler EM Schouten JN Hansen BE van Rooij FJ Hofman A Stricker BH. Prevalence and risk factors of non-alcoholic fatty liver disease in the elderly: results from the rotterdam study. J Hepatol. (2012) 57:1305–11. doi: 10.1016/j.jhep.2012.07.028 22871499

[35] Noureddin M Yates KP Vaughn IA Neuschwander-Tetri BA Sanyal AJ McCullough A. Clinical and histological determinants of nonalcoholic steatohepatitis and advanced fibrosis in elderly patients. Hepatology. (2013) 58:1644–54. doi: 10.1002/hep.26465 PMC3760979 23686698

[36] Mowat AM. Anatomical basis of tolerance and immunity to intestinal antigens. Nat Rev Immunol. (2003) 3:331–41. doi: 10.1038/nri1057 12669023

[37] Thevaranjan N Puchta A Schulz C Naidoo A Szamosi JC Verschoor CP. Age-associated microbial dysbiosis promotes intestinal permeability, systemic inflammation, and macrophage dysfunction. Cell Host Microbe. (2017) 21:455–66 e4. doi: 10.1016/j.chom.2017.03.002 28407483 PMC5392495

[38] Noh JY Herrera M Patil BS Tan XD Wright GA Sun Y. The expression and function of growth hormone secretagogue receptor in immune cells: A current perspective. Exp Biol Med (Maywood). (2022) 247:2184–91. doi: 10.1177/15353702221121635 PMC9899990 36151745

[39] Kline KA Bowdish DME. Infection in an aging population. Curr Opin Microbiol. (2016) 29:63–7. doi: 10.1016/j.mib.2015.11.003 26673958

[40] Glynn JR Moss PAH. Systematic analysis of infectious disease outcomes by age shows lowest severity in school-age children. Sci Data. (2020) 7:329. doi: 10.1038/s41597-020-00668-y 33057040 PMC7566589

[41] Parmigiani A Alcaide ML Freguja R Pallikkuth S Frasca D Fischl MA. Impaired antibody response to influenza vaccine in hiv-infected and uninfected aging women is associated with immune activation and inflammation. PloS One. (2013) 8:e79816. doi: 10.1371/journal.pone.0079816 24236161 PMC3827419

[42] Muyanja E Ssemaganda A Ngauv P Cubas R Perrin H Srinivasan D. Immune activation alters cellular and humoral responses to yellow fever 17d vaccine. J Clin Invest. (2014) 124:3147–58. doi: 10.1172/JCI75429 PMC4071376 24911151

[43] Linehan E Fitzgerald DC. Ageing and the immune system: focus on macrophages. Eur J Microbiol Immunol (Bp). (2015) 5:14–24. doi: 10.1556/EuJMI-D-14-00035 25883791 PMC4397845

[44] Samson LD Boots AMH Verschuren WMM Picavet HSJ Engelfriet P Buisman A-M. Frailty is associated with elevated crp trajectories and higher numbers of neutrophils and monocytes. Exp Gerontology. (2019) 125:110674. doi: 10.1016/j.exger.2019.110674 31336145

[45] Ong S-M Hadadi E Dang T-M Yeap W-H Tan CT-Y Ng T-P. The pro-inflammatory phenotype of the human non-classical monocyte subset is attributed to senescence. Cell Death Dis. (2018) 9:266. doi: 10.1038/s41419-018-0327-1 29449647 PMC5833376

[46] Tran L Greenwood-Van Meerveld B. Age-associated remodeling of the intestinal epithelial barrier. J Gerontol A Biol Sci Med Sci. (2013) 68:1045–56. doi: 10.1093/gerona/glt106 PMC3738030 23873964

[47] Ragonnaud E Biragyn A. Gut microbiota as the key controllers of “Healthy” Aging of elderly people. Immun Ageing. (2021) 18:2. doi: 10.1186/s12979-020-00213-w 33397404 PMC7784378

[48] Feldman N Rotter-Maskowitz A Okun E. Damps as mediators of sterile inflammation in aging-related pathologies. Ageing Res Rev. (2015) 24:29–39. doi: 10.1016/j.arr.2015.01.003 25641058

[49] Kim K-A Jeong J-J Yoo S-Y Kim D-H. Gut microbiota lipopolysaccharide accelerates inflamm-aging in mice. BMC Microbiol. (2016) 16:9. doi: 10.1186/s12866-016-0625-7 26772806 PMC4715324

[50] Bosco N Noti M. The aging gut microbiome and its impact on host immunity. Genes Immun. (2021) 22:289–303. doi: 10.1038/s41435-021-00126-8 PMC8054695 33875817

[51] Noh JY Wu CS DeLuca JAA Devaraj S Jayaraman A Alaniz RC. Novel role of ghrelin receptor in gut dysbiosis and experimental colitis in aging. Int J Mol Sci. (2022) 23:2219. doi: 10.3390/ijms23042219 35216335 PMC8875592

[52] Mishra J Quazi S Kumar N. Increased severity of high molecular weight dss induced colitis in aged mice: P-196. Inflammatory Bowel Dis. (2011) 17:S70–S1. doi: 10.1097/00054725-201112002-00230

[53] Albert EJ Marshall JS. Aging in the absence of tlr2 is associated with reduced ifn-gamma responses in the large intestine and increased severity of induced colitis. J Leukoc Biol. (2008) 83:833–42. doi: 10.1189/jlb.0807557 18223102

[54] Jura M Kozak LP. Obesity and related consequences to ageing. Age (Dordr). (2016) 38:23. doi: 10.1007/s11357-016-9884-3 26846415 PMC5005878

[55] Lee JH Fang C Li X Wu CS Noh JY Ye X. Ghs-R suppression in adipose tissues protects against obesity and insulin resistance by regulating adipose angiogenesis and fibrosis. Int J Obes (Lond). (2021) 45:1565–75. doi: 10.1038/s41366-021-00820-7 PMC8238886 33903722

[56] Singanayagam A Triantafyllou E. Macrophages in chronic liver failure: diversity, plasticity and therapeutic targeting. Front Immunol. (2021) 12:661182. doi: 10.3389/fimmu.2021.661182 33868313 PMC8051585

[57] Liang W Qi Y Yi H Mao C Meng Q Wang H. The roles of adipose tissue macrophages in human disease. Front Immunol. (2022) 13:908749. doi: 10.3389/fimmu.2022.908749 35757707 PMC9222901

[58] Kunz HE Hart CR Gries KJ Parvizi M Laurenti M Dalla Man C. Adipose tissue macrophage populations and inflammation are associated with systemic inflammation and insulin resistance in obesity. Am J Physiol Endocrinol Metab. (2021) 321:E105–E21. doi: 10.1152/ajpendo.00070.2021 PMC8321823 33998291

[59] Cruz AF Rohban R Esni F. Macrophages in the pancreas: villains by circumstances, not necessarily by actions. Immun Inflammation Dis. (2020) 8:807–24. doi: 10.1002/iid3.345 PMC7654401 32885589

[60] Palmer AK Xu M Zhu Y Pirtskhalava T Weivoda MM Hachfeld CM. Targeting senescent cells alleviates obesity-induced metabolic dysfunction. Aging Cell. (2019) 18:e12950. doi: 10.1111/acel.12950 30907060 PMC6516193

[61] Hopkin SJ Pezhman L Begum J Kavanagh D McGettrick HM Iqbal AJ. Aging modulates homeostatic leukocyte trafficking to the peritoneal cavity in a sex-specific manner. J Leukoc Biol. (2023) 114:301–14. doi: 10.1093/jleuko/qiad053 PMC10533226 37309034

[62] Hopkin S Lord JM Chimen M. Dysregulation of leukocyte trafficking in ageing: causal factors and possible corrective therapies. Pharmacol Res. (2021) 163:105323. doi: 10.1016/j.phrs.2020.105323 33276099

[63] Bloomer SA Moyer ED. Hepatic macrophage accumulation with aging: cause for concern? Am J Physiol Gastrointest Liver Physiol. (2021) 320:G496–505. doi: 10.1152/ajpgi.00286.2020 33470190

[64] Covarrubias AJ Kale A Perrone R Lopez-Dominguez JA Pisco AO Kasler HG. Senescent cells promote tissue nad(+) decline during ageing via the activation of cd38(+) macrophages. Nat Metab. (2020) 2:1265–83. doi: 10.1038/s42255-020-00305-3 PMC7908681 33199924

[65] Matacchione G Perugini J Di Mercurio E Sabbatinelli J Prattichizzo F Senzacqua M. Senescent macrophages in the human adipose tissue as a source of inflammaging. Geroscience. (2022) 44:1941–60. doi: 10.1007/s11357-022-00536-0 PMC9616990 35247131

[66] Santoro A Bientinesi E Monti D. Immunosenescence and inflammaging in the aging process: age-related diseases or longevity? Ageing Res Rev. (2021) 71:101422. doi: 10.1016/j.arr.2021.101422 34391943

[67] Kim G Chen Z Li J Luo J Castro-Martinez F Wisniewski J. Gut-liver axis calibrates intestinal stem cell fitness. Cell. (2024) 187:914–30. doi: 10.1016/j.cell.2024.01.001 PMC10923069 38280375

[68] Brubaker AL Rendon JL Ramirez L Choudhry MA Kovacs EJ. Reduced neutrophil chemotaxis and infiltration contributes to delayed resolution of cutaneous wound infection with advanced age. J Immunol. (2013) 190:1746–57. doi: 10.4049/jimmunol.1201213 PMC3563860 23319733

[69] Wenisch C Patruta S Daxböck F Krause R Hörl W. Effect of age on human neutrophil function. J Leukoc Biol. (2000) 67:40–5. doi: 10.1002/jlb.67.1.40 10647996

[70] Shi C Jia T Mendez-Hohl TM Serbina NV Lipuma L. Bone marrow mesenchymal stem and progenitor cells induce monocyte emigration in response to circulating toll-like receptor ligands. Immunity. (2011) 34:590–601. doi: 10.1016/j.immuni.2011.02.016 21458307 PMC3081416

[71] Tak T van Groenendael R Pickkers P Koenderman L. Monocyte subsets are differentially lost from the circulation during acute inflammation induced by human experimental endotoxemia. J Innate Immun. (2017) 9:464–74. doi: 10.1159/000475665 PMC6738874 28641299

[72] Patel AA Zhang Y Fullerton JN Boelen L Rongvaux A Maini AA. The fate and lifespan of human monocyte subsets in steady state and systemic inflammation. J Exp Med. (2017) 214:1913–23. doi: 10.1084/jem.20170355 PMC5502436 28606987

[73] Thaler B Hohensinner PJ Krychtiuk KA Matzneller P Koller L Brekalo M. Differential *in vivo* activation of monocyte subsets during low-grade inflammation through experimental endotoxemia in humans. Sci Rep. (2016) 6:30162. doi: 10.1038/srep30162 27444882 PMC4957086

[74] Silvin A Chapuis N Dunsmore G Goubet AG Dubuisson A Derosa L. Elevated calprotectin and abnormal myeloid cell subsets discriminate severe from mild covid-19. Cell. (2020) 182:1401–18.e18. doi: 10.1016/j.cell.2020.08.002 32810439 PMC7405878

[75] van der Laan AM Ter Horst EN Delewi R Begieneman MP Krijnen PA Hirsch A. Monocyte subset accumulation in the human heart following acute myocardial infarction and the role of the spleen as monocyte reservoir. Eur Heart J. (2014) 35:376–85. doi: 10.1093/eurheartj/eht331 PMC3916776 23966310

[76] Wolf AA Yanez A Barman PK Goodridge HS. The ontogeny of monocyte subsets. Front Immunol. (2019) 10:1642. doi: 10.3389/fimmu.2019.01642 31379841 PMC6650567

[77] Netea MG Dominguez-Andres J Barreiro LB Chavakis T Divangahi M Fuchs E. Defining trained immunity and its role in health and disease. Nat Rev Immunol. (2020) 20:375–88. doi: 10.1038/s41577-020-0285-6 PMC7186935 32132681

[78] Franceschi C Salvioli S Garagnani P de Eguileor M Monti D Capri M. Immunobiography and the heterogeneity of immune responses in the elderly: A focus on inflammaging and trained immunity. Front Immunol. (2017) 8:982. doi: 10.3389/fimmu.2017.00982 28861086 PMC5559470

[79] Zubair K You C Kwon G Kang K. Two faces of macrophages: training and tolerance. Biomedicines. (2021) 9:1596. doi: 10.3390/biomedicines9111596 34829825 PMC8615871

[80] Nomura F Akashi S Sakao Y Sato S Kawai T Matsumoto M. Cutting edge: endotoxin tolerance in mouse peritoneal macrophages correlates with down-regulation of surface toll-like receptor 4 expression. J Immunol. (2000) 164:3476–9. doi: 10.4049/jimmunol.164.7.3476 10725699

[81] Divangahi M Aaby P Khader SA Barreiro LB Bekkering S Chavakis T. Trained immunity, tolerance, priming and differentiation: distinct immunological processes. Nat Immunol. (2021) 22:2–6. doi: 10.1038/s41590-020-00845-6 33293712 PMC8020292

[82] Gillen J Ondee T Gurusamy D Issara-Amphorn J Manes NP Yoon SH. Lps tolerance inhibits cellular respiration and induces global changes in the macrophage secretome. Biomolecules. (2021) 11:164. doi: 10.3390/biom11020164 33513762 PMC7918892

[83] Bauer M Weis S Netea MG Wetzker R. Remembering pathogen dose: long-term adaptation in innate immunity. Trends Immunol. (2018) 39:438–45. doi: 10.1016/j.it.2018.04.001 29716792

[84] Seeley JJ Ghosh S. Molecular mechanisms of innate memory and tolerance to lps. J Leukoc Biol. (2017) 101:107–19. doi: 10.1189/jlb.3MR0316-118RR 27780875

[85] Ochando J Mulder WJM Madsen JC Netea MG Duivenvoorden R. Trained immunity - basic concepts and contributions to immunopathology. Nat Rev Nephrol. (2023) 19:23–37. doi: 10.1038/s41581-022-00633-5 36253509 PMC9575643

[86] Geng H Bu HF Liu F Wu L Pfeifer K Chou PM. In inflamed intestinal tissues and epithelial cells, interleukin 22 signaling increases expression of H19 long noncoding rna, which promotes mucosal regeneration. Gastroenterology. (2018) 155:144–55. doi: 10.1053/j.gastro.2018.03.058 PMC6475625 29621481

[87] Raduolovic K Mak’Anyengo R Kaya B Steinert A Niess JH. Injections of Lipopolysaccharide into Mice to Mimic Entrance of Microbial-Derived Products after Intestinal Barrier Breach. J Vis Exp. (2018) 135:e57610. doi: 10.3791/57610 PMC6101093 29782025

[88] Marquez EJ Chung CH Marches R Rossi RJ Nehar-Belaid D Eroglu A. Sexual-dimorphism in human immune system aging. Nat Commun. (2020) 11:751. doi: 10.1038/s41467-020-14396-9 32029736 PMC7005316

[89] Klein SL Flanagan KL. Sex differences in immune responses. Nat Rev Immunol. (2016) 16:626–38. doi: 10.1038/nri.2016.90 27546235

[90] Jaillon S Berthenet K Garlanda C. Sexual dimorphism in innate immunity. Clin Rev Allergy Immunol. (2019) 56:308–21. doi: 10.1007/s12016-017-8648-x 28963611

[91] Sampathkumar NK Bravo JI Chen Y Danthi PS Donahue EK Lai RW. Widespread sex dimorphism in aging and age-related diseases. Hum Genet. (2020) 139:333–56. doi: 10.1007/s00439-019-02082-w PMC7031050 31677133

[92] Dunn SE Perry WA Klein SL. Mechanisms and consequences of sex differences in immune responses. Nat Rev Nephrol. (2024) 20:37–55. doi: 10.1038/s41581-023-00787-w 37993681

[93] So J Tai AK Lichtenstein AH Wu D Lamon-Fava S. Sexual dimorphism of monocyte transcriptome in individuals with chronic low-grade inflammation. Biol Sex Differ. (2021) 12:43. doi: 10.1186/s13293-021-00387-y 34321081 PMC8320037

[94] Li K Xu W Guo Q Jiang Z Wang P Yue Y. Differential macrophage polarization in male and female balb/C mice infected with coxsackievirus B3 defines susceptibility to viral myocarditis. Circ Res. (2009) 105:353–64. doi: 10.1161/CIRCRESAHA.109.195230 19608981

[95] Trial J Diaz Lankenau R Angelini A Tovar Perez JE Taffet GE Entman ML. Treatment with a dc-sign ligand reduces macrophage polarization and diastolic dysfunction in the aging female but not male mouse hearts. Geroscience. (2021) 43:881–99. doi: 10.1007/s11357-020-00255-4 PMC8110645 32851570

[96] Kratofil RM Shim HB Shim R Lee WY Labit E Sinha S. A monocyte-leptin-angiogenesis pathway critical for repair post-infection. Nature. (2022) 609:166–73. doi: 10.1038/s41586-022-05044-x 35948634

[97] Wynn TA Vannella KM. Macrophages in tissue repair, regeneration, and fibrosis. Immunity. (2016) 44:450–62. doi: 10.1016/j.immuni.2016.02.015 PMC4794754 26982353

[98] Phillipson M Kubes P. The healing power of neutrophils. Trends Immunol. (2019) 40:635–47. doi: 10.1016/j.it.2019.05.001 31160208

[99] Wang J Kubes P. A reservoir of mature cavity macrophages that can rapidly invade visceral organs to affect tissue repair. Cell. (2016) 165:668–78. doi: 10.1016/j.cell.2016.03.009 27062926

[100] Duong L Radley HG Lee B Dye DE Pixley FJ Grounds MD. Macrophage function in the elderly and impact on injury repair and cancer. Immun Ageing. (2021) 18:4. doi: 10.1186/s12979-021-00215-2 33441138 PMC7805172

[101] Coffman JA Rieger S Rogers AN Updike DL Yin VP. Comparative biology of tissue repair, regeneration and aging. NPJ Regener Med. (2016) 1:16003–. doi: 10.1038/npjregenmed.2016.3

[102] Yue Z Nie L Zhang P Chen Q Lv Q Wang Q. Tissue-resident macrophage inflammaging aggravates homeostasis dysregulation in age-related diseases. Cell Immunol. (2021) 361:104278. doi: 10.1016/j.cellimm.2020.104278 33445052

[103] Li Z Zhang Z Ren Y Wang Y Fang J Yue H. Aging and age-related diseases: from mechanisms to therapeutic strategies. Biogerontology. (2021) 22:165–87. doi: 10.1007/s10522-021-09910-5 PMC7838467 33502634

[104] Aroda VR Edelstein SL Goldberg RB Knowler WC Marcovina SM Orchard TJ. Long-term metformin use and vitamin B12 deficiency in the diabetes prevention program outcomes study. J Clin Endocrinol Metab. (2016) 101:1754–61. doi: 10.1210/jc.2015-3754 PMC4880159 26900641

