# Supplementary material for: Innate immunity in peripheral tissues is differentially impaired under normal and endotoxic conditions in aging
Source: Front Immunol. 2024 Aug 16;15:1357444. doi: 10.3389/fimmu.2024.1357444 (PMC11361940; doi:10.3389/fimmu.2024.1357444)
Supplement: Supplementary file 1 [file Datasheet1.docx]

Supplementary Material

**Innate immunity in peripheral tissues is differentially impaired under normal and endotoxic conditions in aging**

Ji Yeon Noh^1^, Hye Won Han^1^, Da Mi Kim^1^, Erin D. Giles^2^, Yuhua Z. Farnell^3^, Gus A. Wright^4^, Yuxiang Sun^1,5*^


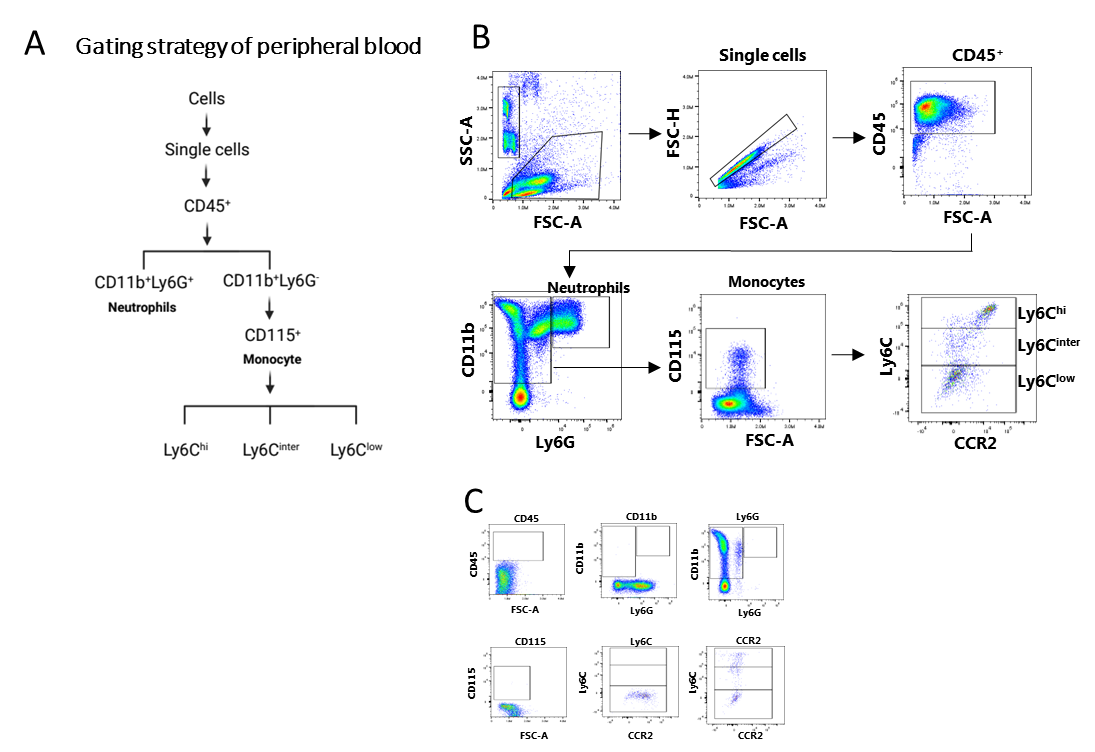
* Correspondence: Yuxiang Sun*: yuxiangs@tamu.edu

**Supplementary Figure 1.** **(A)** Gating strategy of peripheral blood **(B)** Representative plots of flow cytometry analysis and **(C)** FMO controls


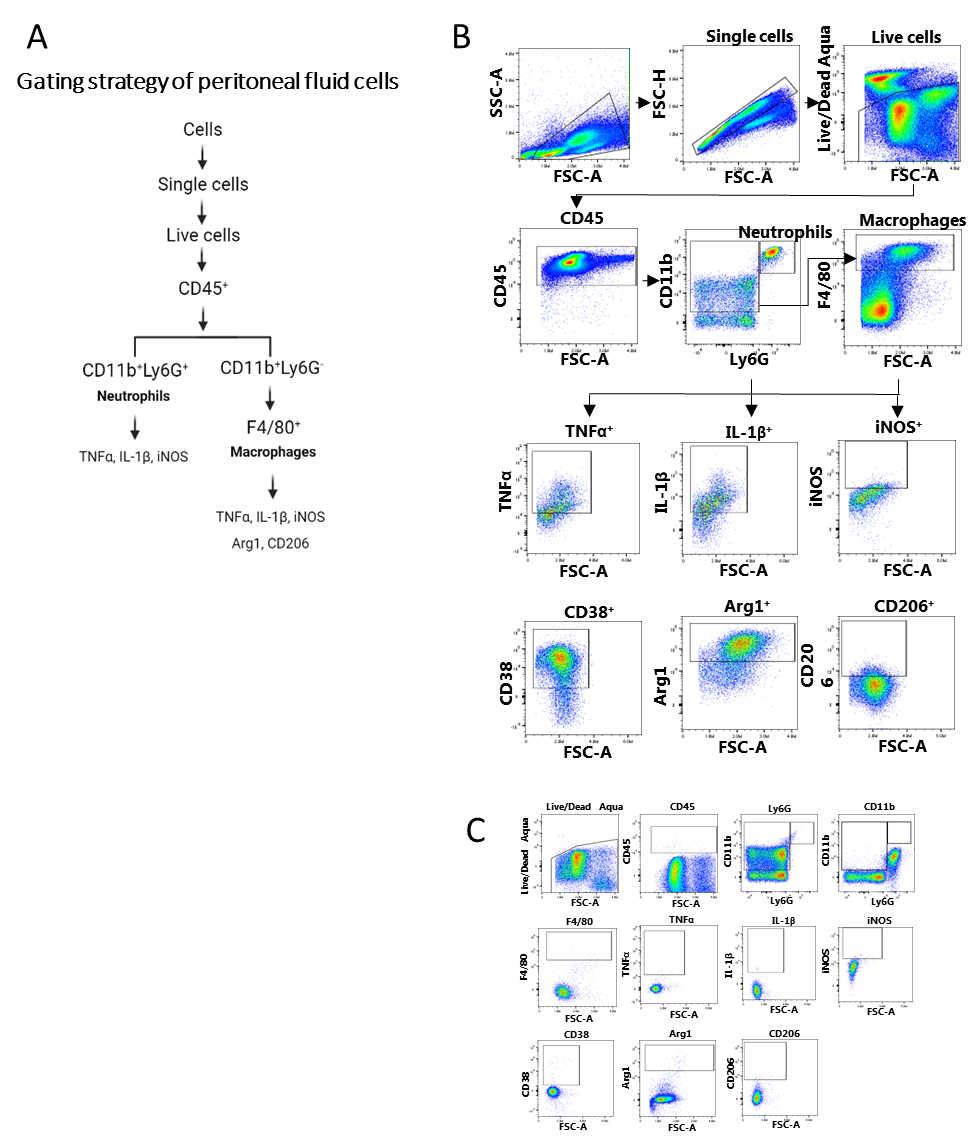


**Supplementary Figure 2.** **(A)** Gating strategy of peritoneal fluid cells **(B)** Representative plots of flow cytometry analysis and **(C)** FMO controls


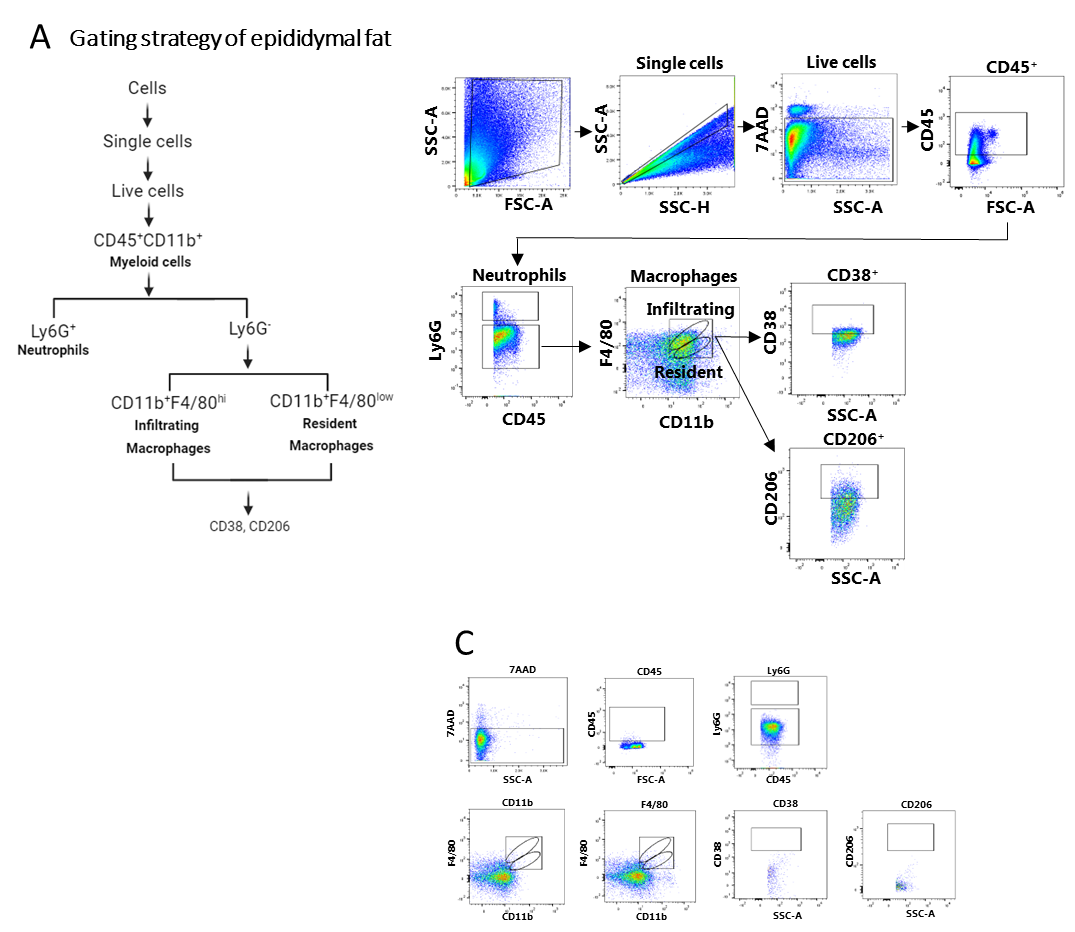


**Supplementary Figure 3.** **(A)** Gating strategy of stromal vascular fraction (SVF) from epididymal fat **(B)** Representative plots of flow cytometry analysis and **(C)** FMO controls

**
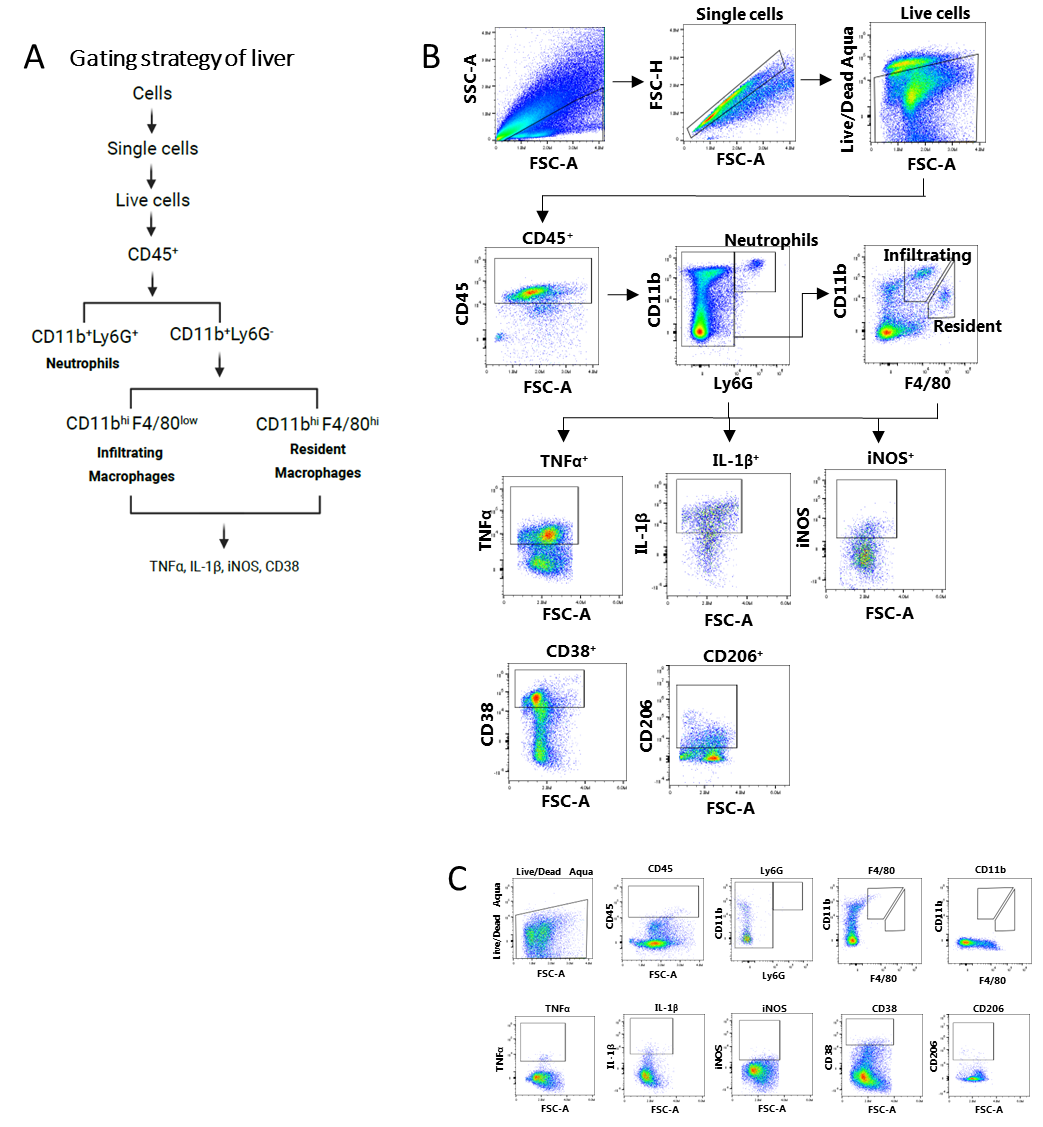
**

**Supplementary Figure 4.** **(A)** Gating strategy of liver **(B)** Representative plots of flow cytometry analysis and **(C)** FMO controls


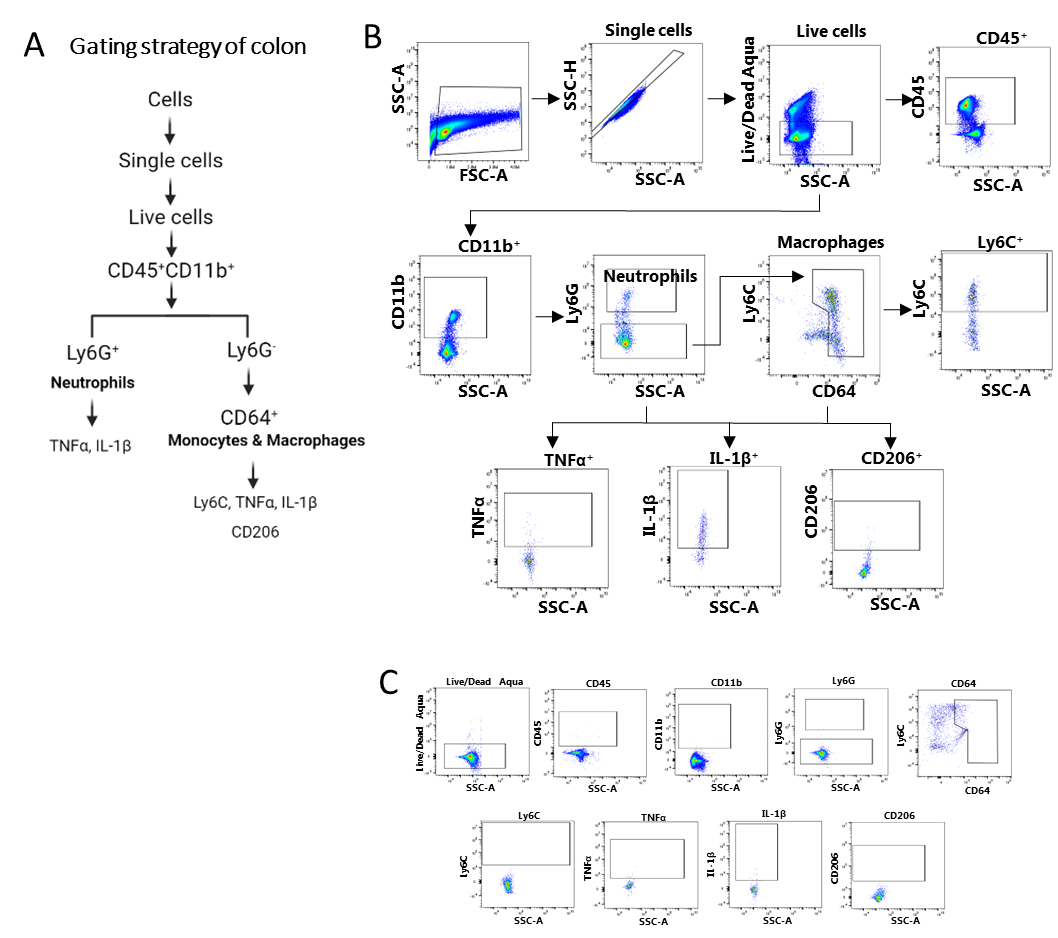


**Supplementary Figure 5.** **(A)** Gating strategy of colon **(B)** Representative plots of flow cytometry analysis and **(C)** FMO controls


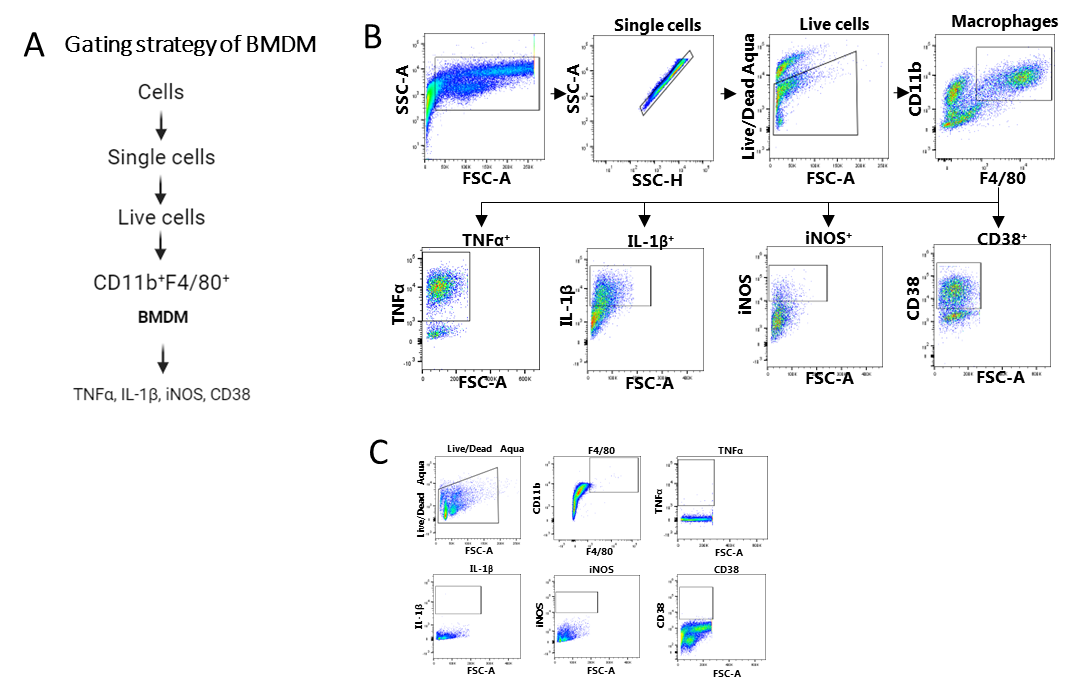


**Supplementary Figure 6.** **(A)** Gating strategy of BMDM **(B)** Representative plots of flow cytometry analysis and **(C)** controls
